# Diagnostic accuracy of multiplex respiratory pathogen panels for influenza or respiratory syncytial virus infections: systematic review and meta-analysis

**DOI:** 10.1186/s12879-022-07766-9

**Published:** 2022-10-13

**Authors:** Sophie Jullien, Felicity Fitzgerald, Suzanne Keddie, Oliver Baerenbold, Quique Bassat, John Bradley, Jane Falconer, Colin Fink, Ruth Keogh, Heidi Hopkins, Marie Voice

**Affiliations:** 1 Barcelona Institute for Global Health (ISGlobal), Hospital Clínic - Universitat de Barcelona, Carrer Rosselló 132, 08036 Barcelona, Spain; 2 Imperial College London, London, UK; 3 London School of Hygiene & Tropical Medicine, London, UK; 4 Centro de Investigação Em Saúde de Manhiça (CISM), Maputo, Mozambique; 5 ICREA, Pg. Lluís Companys 23, 08010 Barcelona, Spain; 6 Pediatrics Department, Hospital Sant Joan de Déu, Universitat de Barcelona, Esplugues, Barcelona, Spain; 7 Consorcio de Investigación Biomédica en Red de Epidemiología y Salud Pública (CIBERESP), Madrid, Spain; 8 Micropathology Ltd., University of Warwick Science Park, Coventry, UK; 9 Department of Life Sciences, University of Warwick, Coventry, UK

**Keywords:** Respiratory syncytial virus, Influenza virus, Diagnosis, Molecular diagnostics, Respiratory infection

## Abstract

Respiratory syncytial virus (RSV) and influenza viruses are important global causes of morbidity and mortality. We evaluated the diagnostic accuracy of the Luminex NxTAG respiratory pathogen panels (RPPs)™ (index) against other RPPs (comparator) for detection of RSV and influenza viruses. Studies comparing human clinical respiratory samples tested with the index and at least one comparator test were included. A random-effect latent class meta-analysis was performed to assess the specificity and sensitivity of the index test for RSV and influenza. Risk of bias was assessed using the QUADAS-2 tool and certainty of evidence using GRADE. Ten studies were included. For RSV, predicted sensitivity was 99% (95% credible interval [CrI] 96–100%) and specificity 100% (95% CrI 98–100%). For influenza A and B, predicted sensitivity was 97% (95% CrI 89–100) and 98% (95% CrI 88–100) respectively; specificity 100% (95% CrI 99–100) and 100% (95% CrI 99–100), respectively. Evidence was low certainty. Although index sensitivity and specificity were excellent, comparators’ performance varied. Further research with clear patient recruitment strategies could ascertain performance across different populations.

*Protocol Registration:* Prospero CRD42021272062.

**Supplementary Information:**

The online version contains supplementary material available at 10.1186/s12879-022-07766-9.

## Background

Respiratory syncytial virus (RSV) and influenza viruses are important causes of global morbidity and mortality. An estimated ~ 33 million episodes of RSV occur annually in children under five years of age, causing at least 3.2 million hospital admissions and 59,600 in-hospital deaths, with an even higher unmeasured community burden in low-resource settings [1–3]. Influenza is estimated to cause up to 650,000 deaths per year, the majority in low-resource settings and in people over 75 years old [4]. However, current estimates suggest that up to 100,000 deaths from influenza occur annually in children under five years old [4, 5]. Post-mortem studies in childhood deaths under the age of five also show a higher than expected burden of these two pathogens [6]. Given that RSV also causes significant mortality in the elderly, these two viruses pose significant health risks throughout the human lifespan [7].

The need for rapid, accurate diagnostics for these pathogens is threefold. Firstly, for the purposes of antimicrobial stewardship: the symptoms of respiratory tract infections are non-specific, and empiric antibiotics are frequently commenced to cover possible bacterial pneumonia [8]. Rapid viral tests can therefore reduce the unnecessary prescription of antibiotics, although viral detection does not exclude bacterial co-infection [9–11]. Secondly, to confirm the specific viral cause of illness and guide commencement (or cessation) of specific antiviral therapy [12]. Finally, rapid diagnostic tests have a crucial role in determining need for infection control prevention.

In recent years there has been a rise in the use of proprietary multiplex respiratory pathogen panels (RPP) in routine clinical setting, using a range of technologies, which have increasingly replaced in-house individual real-time polymerase chain reaction (RT-PCR) assays for clinical diagnostics [13]. This follows improved turn-around time, reduced number of manual steps in the laboratory, and the multiplexing of several pathogens within a single panel, alongside a continuous evolution of regional regulatory standards [14, 15]. Luminex NxTAG RPP™ is one such panel, offering high throughput of up to a hundred samples per run with the potential to test for up to 21 viral and bacterial pathogen genes simultaneously in each sample, improving both turn-around time and cost-effectiveness [16].

The Febrile Illness Evaluation in a Broad Range of Endemicities (FIEBRE) is a prospective observational study of the infectious causes of fever at four sites in Africa and Asia, collecting data and samples from inpatients, outpatients and community controls [17]. FIEBRE focuses on illnesses deemed preventable or treatable; respiratory pathogens of interest include RSV and influenza viruses. The Luminex NxTAG RPP™ on respiratory samples was chosen as the reference standard for detecting these infections in the FIEBRE study. Firstly, it is CE marked for in vitro diagnostic (IVD) use and is internally verified by the assigned United Kingdom Accreditation Service accredited laboratory [18]. Secondly, its high-throughput platform allows for 96 samples to be analysed per run, multiplexing 21 genes (hence testing for up to 21 pathogens at once). In this systematic review and meta-analysis, we aimed to evaluate the diagnostic accuracy of the Luminex NxTAG RPP™ in comparison to other RPP for the detection of RSV and influenza viruses in respiratory samples. This systematic review is part of a series conducted by the FIEBRE research team, with the purpose of determining the accuracy of reference tests used to diagnose infectious causes of fever.

## Methods

### Inclusion criteria

We included observational and interventional studies that reported findings of the Luminex NxTAG RPP™ assay performed to detect influenza A/B viruses and RSV in respiratory samples from children (aged 2 months and older) and adults attending healthcare settings. We first intended to include studies testing for the Luminex NxTAG RPP™ assay in patients with reported or documented fever, but we found no such study. We broadened our inclusion criteria, therefore, to studies when patients were tested with the Luminex NxTAG RPP™ assay (index test I) and at least one other RPP as comparator (C). We excluded studies describing in vitro identification of viruses as opposed to detection in clinical samples and studies that did not provide data from which we could extract a binary classification table (I + /C + , I−/C + , I−/C− and I + /C−).

### Search methods

An experienced library information specialist (JF) compiled a search strategy in the OvidSP Medline database. The search strategy included strings of terms, synonyms and controlled vocabulary terms (where available) to reflect two concepts: respiratory tract infections, specifically RSV or influenza, and Luminex NxTAG RPP™. The search was limited to papers published from January 2015, when Luminex NxTAG RPP™ assay was commercialized. No other search filters or limits were added. The agreed OvidSP Medline search was adapted for each database to incorporate database-specific syntax and controlled vocabularies (Additional file 1: Annex S1). We searched the following databases on 22 September 2020: OvidSP Medline, OvidSP Embase, OvidSP Global Health, Wiley Cochrane Central Register of Controlled Trials, Clarivate Analytics Web of Science, Elsevier Scopus, Ebesco Africa-Wide Information, WHO LILACS and WHO Global Index Medicus (Additional file 1: Annex S2). We imported all citations identified by our searches into EndNote X9 software and identified and removed duplicates [19]. To identify additional eligible studies, we hand-searched the reference lists of relevant manuscripts and contacted the Luminex manufacturer.

### Study selection

Two reviewers (SJ, FF) selected studies independently and in duplicate using the online tool CADIMA [20]. We performed the initial eligibility assessment of titles and abstracts identified by the search strategy, using the pre-determined eligibility criteria. We retrieved full-text copies of potentially eligible reports and contacted researchers for further information when needed. We resolved disagreements through discussion and excluded reports not meeting criteria.

### Data collection and methodological quality assessment

We piloted the data extraction form and quality assessment on two studies. For each study, using the finalized data extraction form, two reviewers (SJ, FF) independently extracted data including study design, methodology, participant and comparator test characteristics, and flow and timing of sample analysis. We contacted study investigators when data reported were unclear or insufficient to produce 2 × 2 tables for I + /C + , I + /C−, I−/C + and I−/C−.

Two independent reviewers (SJ, FF) evaluated the quality of each study using the quality assessment tool for diagnostic accuracy studies (QUADAS-2), which assesses both the risk of bias and applicability to the review question for four domains: patient selection, index test, reference standard (renamed as comparator test for this review) and the flow and timing of patients through the study [21]. We resolved disagreements by discussion.

### Statistical analysis and data synthesis

We extracted for each study the performance results for the Luminex NxTAG RPP™ test and the comparator test into a 2 × 2 table. Where a study used multiple comparator tests, we created a 2 × 2 table for each comparator. Within the statistical analyses, test results from discrepancy resolution (results from a third test when results from the index and comparator tests differed) were not included [22].

We implemented a Bayesian random-effect latent class meta-analysis, which is an extension to the hierarchical summary receiver operating characteristic (HSROC) Model [23], to estimate the sensitivity and specificity of Luminex NxTAG RPP™. This approach takes into account within- and between-study variation as well as accounting for multiple imperfect comparator tests. The model allows us to relax the assumption that, conditional on disease status, tests on the same individual are independent. Inference is done on the estimated mean sensitivity and specificity across studies, i.e. pooled sensitivity/specificity, and the predicted diagnostic accuracy in an out-of-sample study, i.e. predicted sensitivity/specificity. For RSV and influenza separately, we present modelled estimates of the Luminex NxTAG RPP™ test sensitivity and specificity within each study along with a single pooled estimate. By assessing the variability within the studies included in the present meta-analysis we are able to predict the sensitivity and specificity of the Luminex NxTAG RPP™ test if it were applied to a future similar population. We present these predicted estimates of Luminex NxTAG RPP™ for RSV and influenza viruses as summary ROC curves, plotting the 95% credible region. The meta-analyses were implemented using Stan in R [24]. A full model specification including the choice of prior distributions and sensitivity analyses can be found in Additional file 1: Annex S3.

We fit separate meta-analyses for RSV and influenza. Within the influenza model we explored heterogeneity between influenza A and influenza B viruses and present pooled estimates by influenza type.

### Assessment of the certainty of the evidence

We assessed the certainty of the evidence using GRADE and GRADEpro GDT software [25–27]. We rated certainty as high, moderate, low, or very low across four domains (risk of bias, indirectness, inconsistency and imprecision). We assessed risk of bias and indirectness by using the QUADAS-2 tool [21]. We explored inconsistency by investigating potential sources of heterogeneity. For imprecision, we considered the width of the Bayesian credible intervals (CrI). We calculated I + /C + , I + /C−, I−/C + and I−/C−, with ranges for these values based on the CrI of the predicted estimates of sensitivity and specificity for prevalences of 5% and 20% of RSV or influenza viruses, and we made judgements on imprecision using these calculations.

The protocol, developed prior to conducting the review, is accessible online (Prospero CRD42021272062) [28].

## Results

We identified 610 potentially eligible studies (Additional file 1: Annex S4). Of these, ten met our selection criteria and were included in the review and meta-analysis (Additional file 1: Fig. S1).

### Study description

The ten studies included are described in Additional file 1: Tables S1–S10 and their key findings in Table 1.

**Table 1:** Summary of characteristics of the studies included in this review

Study ID	Setting	Participants	Samples	n	Comparator tests
Beckmann 2016	Switzerland	Children and adults Symptoms and fever not reported	NPS (199), BAL (76), others (7)	282	RespiFinder-221^a^
Brotons 2016	Spain	< 18 years with ALRI Fever not reported	NPA	320	Anyplex II RV16 assay^a,b,c^
Chan 2017	China	Children and adults with ARI Fever not reported	NPA	133	RT-PCR **AND** DFA^a^
Chen 2016	China	Patients with ARI Fever not reported	NPS	284	FilmArray RP^a,d,e^
Esposito 2016	Italy	Children with ARI in PICU and children with pneumonia by *M. pneumoniae*	NPS	185	xTAG RVP FAST v2^a,c^ RT-PCR
Gonsalves 2019	USA, Canada	Children and adults with ARI Fever not reported	NPW	2132	xTAG RVP^a,c^ **OR** bidirectional sequencing
Lee 2017	Singapore	Not reported	Respiratory samples	142	xTAG RVP FAST v2^a,c^
Locher 2019	Canada	Adults, mostly immunocompromised and with underlying chronic lung conditions, with ARI	Bronchoscopy collected samples	133	FilmArray RP^a,d,e^
Sails 2017	United Kingdom	“Symptomatic” Other characteristics not reported	NPS (122), throat swabs (53), endotracheal (47), BAL (17), others (122)	314	In-house multiplex RT-PCR panel
Tang 2016	USA	Patients with respiratory symptoms Fever not reported	NPS	404	FilmArray RP^a,d,e^

*ALRI* acute lower respiratory infection, *ARI* acute respiratory infection, *BAL* broncho-alveolar lavage, *DFA* direct immunofluorescence, *n* sample size, *NPA* nasopharyngeal aspirate, *NPS* nasopharyngeal swabs, *RP* respiratory panel; *RVP* respiratory virus panel

^a^Complies with CE-IVD regulations

^b^Complies with Canadian Department of Health regulations

^c^Complies with Korea Food and Drug Administration regulations

^d^Complies with the United States Food and Drug Administration regulations

^e^Complies with Therapeutic Goods Administration regulations

The studies included data from 4329 samples. Samples were collected from children and adults in three studies [29–31], children only in two [32, 33], adults only in one [34], and age was not specified in the remaining studies [35–38]. For seven studies, participants were recruited if they presented with symptoms suggestive of acute lower respiratory infection; this was not clearly stated for the remaining three studies [29, 36, 37]. No study specified fever as an inclusion criterion, nor reported the proportion of participants with fever. Patients were selected from a paediatric intensive care unit in one study [33]. In another, samples were collected from mostly immunocompromised patients with underlying chronic lung conditions [34]. Luminex NxTAG RPP™ was performed on 2132 nasopharyngeal washings [31], 1194 nasopharyngeal swabs [29, 33, 35, 37, 38], 453 nasopharyngeal aspirates [30, 32] and other respiratory samples [29, 34, 36, 37].

The studies compared Luminex NxTAG RPP™ with various comparator tests including BioFire FilmArray Respiratory Panel [34, 35, 38], xTAG Respiratory Virus Panel Fast Assay v2 [33, 36], xTAG Respiratory Virus Panel [31], RespiFinder-221 [29], Anyplex II RV16 assay [32], RT-PCR [33, 37], and bidirectional sequencing [31]. In one study, the comparator consisted of the combination of RT-PCR and direct immunofluorescence [30]. Another used either xTAG Respiratory Virus Panel or RT-PCR as comparator, without providing disaggregated data [31].

### Methodological quality of included studies

**Fig. 1:**
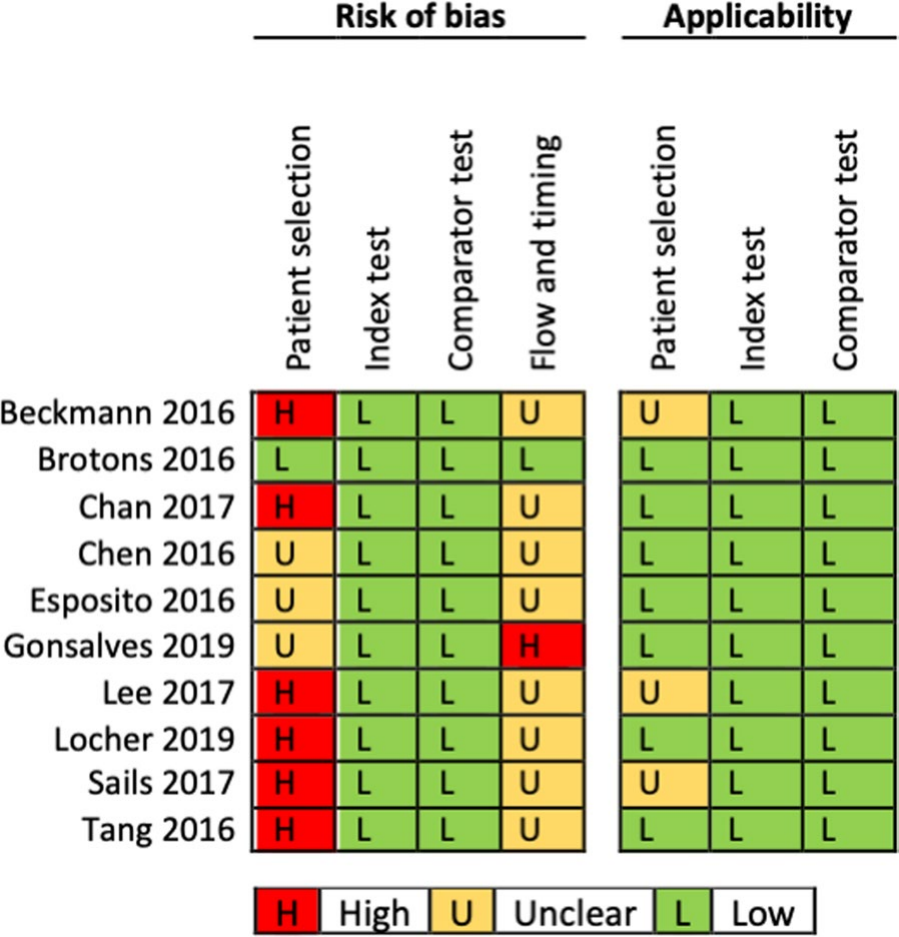
Risk of bias and applicability concerns in the accuracy of Luminex NxTAGG RPP™ for the detection of RSV and influenza viruses in respiratory samples

See Additional file 1: Tables S1–S10 for the assessment of the methodological quality of each study included. Figure 1 summarizes the risk of bias and applicability concerns, describing our judgements about each domain for each included study.

In the patient selection domain, we judged six studies at high risk of bias, because recruitment of participants was not consecutive or random but planned after the test was performed on patients selected by physician (e.g. respiratory symptoms) with no clear inclusion and exclusion criteria [29, 30, 34, 36–38]. Regarding applicability, we rated three studies as ‘unclear’ as inclusion criteria were not recorded by the investigators [29, 36, 37] and considered the remaining seven studies to match the review question.

In the index test domain, we considered all ten studies at low risk of bias, because Luminex NxTAG RPP™ was interpreted without the knowledge of the results of the comparator, and because we judged that knowing the result of the comparator was at very low risk of introducing bias due to the test characteristics.

In the comparator test domain, we judged all the studies to be at low risk of bias because knowing the finding of the index test is at low risk of introducing bias in the interpretation of the comparator tests, due to their intrinsic characteristics.

In the flow and timing domain, we considered eight studies at unclear risk of bias because samples were stored for a long or unclear duration between the performance of the index and comparator tests [29, 30, 33–38]. We considered one study at high risk of bias because investigators did not use the same comparator for all the samples [31], and one study at low risk of bias because index and comparator tests were performed on the same sample collected prospectively [32].

### Findings

#### Luminex NxTAG RPP™ for detection of RSV

The ten studies reported findings of Luminex NxTAG RPP™ and at least one other RPP for detecting RSV (Additional file 1: Table S11). Two studies reported data separately for RSV-A and RSV-B such that it was not possible to pool data for RSV-A or RSV-B, as possible co-infection was not reported [30, 32]. Six studies provided disaggregated findings for RSV-A and RSV-B (Additional file 1: Table S12) [29–33, 37].

**Fig. 2:**
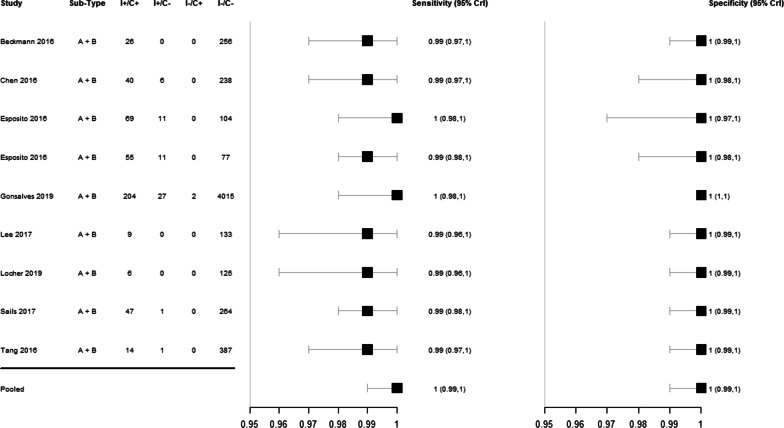
Sensitivity and specificity estimations for the Luminex NxTAG RPP™ versus comparator diagnostic test panels for detecting RSV in respiratory samples with their 95% credible intervals (CrI). The forest plot includes two entries for the Esposito 2016 study, one for each comparator included in that study [33]

The studies included had estimated mean sensitivities ranging from 99 to 100% and specificities of 100% (Additional file 1: Table S11, Fig. 2).

**Fig. 3:**
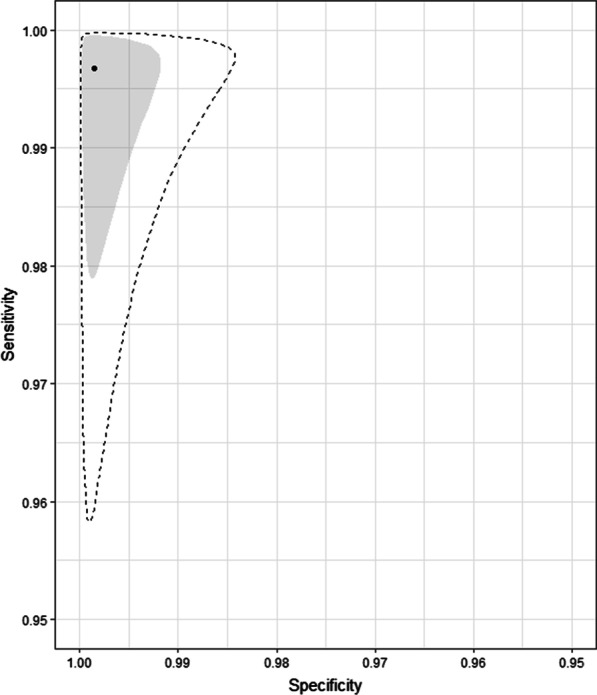
Pooled (shaded) and predicted (dashed) credible regions of Luminex NxTAG RPP™ for detecting RSV in respiratory samples

The pooled sensitivity of Luminex NxTAG RPP™ was 100% (95% CrI 99–100) and pooled specificity was 100% (95% CrI 99–100). Predicted sensitivity was 99% (95% CrI 96–100, 8 studies, 527 samples; low certainty evidence) and predicted specificity was 100% (95% CrI 98–100, 8 studies, 5601 samples; low certainty evidence) (Table 2; Fig. 3).

**Table 2:** Summary of findings for diagnostic accuracy of Luminex NxTAG RPP™ for the diagnosis of RSV

Outcome	Effect per 1000 patients tested		№ of studies (№ of samples)	Test accuracy certainty of evidence
	Pre-test probability of 5%	Pre-test probability of 20%		
Index and comparator tests positive (I + /C +) (patients with RSV infection)	50 (48 to 50)	198 (192 to 200)	8 studies 527 samples	⊕ ⊕ ⊝ ⊝^a^ LOW
Index test negative, comparator positive (I−/C +) (patients incorrectly classified as not having RSV infection)	0 (0 to 2)	2 (0 to 8)		
Index and comparator tests negative (I−/C−) (patients without RSV infection)	950 (931 to 950)	800 (784 to 800)	8 studies 5601 samples	⊕ ⊕ ⊝ ⊝^a^ LOW
Index test positive, comparator negative (I + /C−) (patients incorrectly classified as having RSV infection)	0 (0 to 19)	0 (0 to 16)		

*C* comparator, *CrI* credible interval, *I* index test, *RPP* respiratory pathogen panel

Patient or population: adults and children with symptoms of acute lower respiratory infection

Setting: worldwide

Index test: Luminex NxTAG RPP™

Comparator tests: other RPP

Predicted sensitivity: 0.99 (95% CrI: 0.96 to 1.00) | Predicted specificity: 1.00 (95% CrI: 0.98 to 1.00)

^a^Downgraded two levels for risk of bias: there is high or unclear risk of bias on the patient selection and flow and timing domains for all included studies. Six studies were planned after the test was performed on patients selected by physician (e.g. respiratory symptoms) with no clear inclusion and exclusion criteria, which is at high risk of introducing bias for evaluating diagnostic test accuracy.

The performance of Luminex NxTAG RPP™ appeared similar for RSV-A and RSV-B (Additional file 1: Fig. S2 and S3) [30, 37].

#### Luminex NxTAG RPP™ for detection of influenza viruses

All except one study [33] reported findings of Luminex NxTAG RPP™ and at least one other RPP for detecting influenza A and influenza B (Additional file 1: Table S13). Seven studies presented disaggregated data for influenza subtypes AH1 and AH3 [29–31, 35–38]. For three of these, we inferred the 2 × 2 table for influenza A from the subtypes data assuming no co-infection [29, 37, 38].

**Fig. 4:**
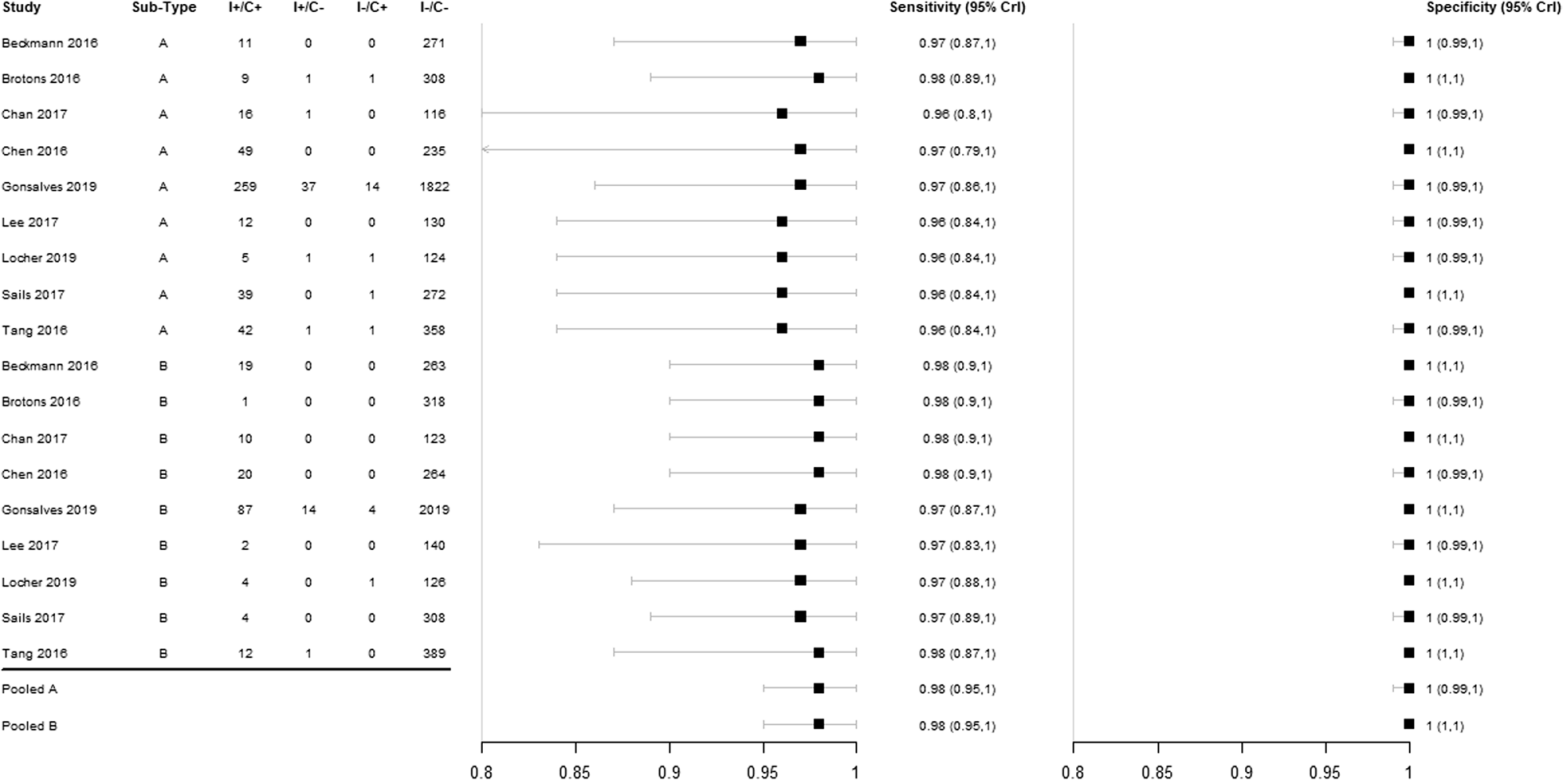
Forest plot of the sensitivity and specificity of Luminex NxTAG RPP™ versus comparator RPP for detecting influenza virus in respiratory samples, with their 95% credible intervals (CrI)

For detection of influenza A virus, mean sensitivity estimates were 96–98% and mean specificity estimates were 100% in all studies (Additional file 1: Table S13, Fig. 4).

**Fig. 5:**
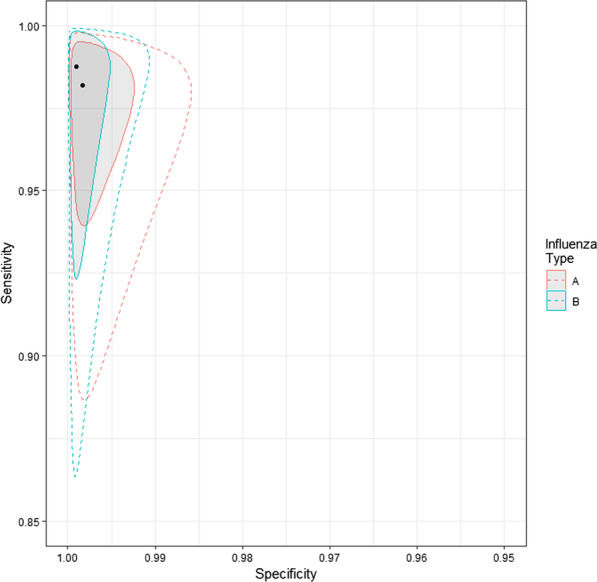
Pooled (shaded) and predicted (dashed) credible regions of Luminex NxTAG RPP™ for detecting influenza viruses in respiratory samples

Pooled sensitivity of Luminex NxTAG RPP™ against comparator tests was 98% (95% CrI 95–100) and pooled specificity was 100% (95% CrI 99–100). Predicted sensitivity was 97% (95% CrI 89–100, 9 studies, 460 samples; low certainty evidence) and predicted specificity was 100% (95% CrI 99–100, 9 studies, 3677 samples; low certainty evidence) (Table 3; Fig. 5).

**Table 3:** Summary of findings for diagnostic accuracy of Luminex NxTAG RPP™ for the diagnosis of influenza A virus

Outcome	Effect per 1000 patients tested		№ of studies (№ of samples)	Test accuracy certainty of evidence
	Pre-test probability of 5%	Pre-test probability of 20%		
Index and comparator tests positive (I + /C +) (patients with influenza A infection)	49 (45 to 50)	194 (178 to 200)	9 studies 460 samples	⊕ ⊕ ⊝ ⊝^a^ LOW
Index test negative, comparator positive (I−/C +) (patients incorrectly classified as not having influenza A infection)	1 (0 to 5)	6 (0 to 22)		
Index and comparator tests negative (I−/C−) (patients without influenza A infection)	950 (941 to 950)	800 (792 to 800)	9 studies 3677 samples	⊕ ⊕ ⊝ ⊝^a^ LOW
Index test positive, comparator negative (I + /C−) (patients incorrectly classified as having influenza A infection)	0 (0 to 9)	0 (0 to 8)		

*C* comparator, *CrI* credible interval, *I* index test, *RPP* respiratory pathogen panel

Patient or population: adults and children with symptoms of acute lower respiratory infection

Setting: worldwide

Index test: Luminex NxTAG RPP™

Comparator tests: other RPP

Predicted sensitivity: 0.97 (95% CrI: 0.89 to 1.00) | Predicted specificity: 1.00 (95% CrI: 0.99 to 1.00)

^a^Downgraded two levels for risk of bias: there is high or unclear risk of bias on the patient selection and flow and timing domains for all included studies. Six studies were planned after the test was performed on patients selected by physician (e.g. respiratory symptoms) with no clear inclusion and exclusion criteria, which is at high risk of introducing bias for evaluating diagnostic test accuracy

For detection of influenza B virus, mean sensitivity estimates ranged from 97 to 98% and mean specificity was 100% in all studies [31] (Table 4, Fig. 4). Pooled sensitivity was 98% (95% CrI 95–100) and pooled specificity was 100% (95% CrI 100–100). Predicted sensitivity was 98% (95% CrI 88–100, 9 studies, 164 participants; low certainty evidence) and predicted specificity was 100% (95% CrI 99–100, 9 studies, 3965 participants; low certainty evidence) (Table 4; Fig. 5).

**Table 4:** Summary of findings for diagnostic accuracy of Luminex NxTAG RPP™ for the diagnosis of influenza B virus

Outcome	Effect per 1000 patients tested		№ of studies (№ of samples)	Test accuracy certainty of evidence
	Pre-test probability of 5%	Pre-test probability of 20%		
Index and comparator tests positive (I + /C +) (patients with influenza B infection)	49 (45 to 50)	194 (178 to 200)	9 studies 164 samples	⊕ ⊕ ⊝ ⊝^a^ LOW
Index test negative, comparator positive (I−/C +) (patients incorrectly classified as not having influenza B infection)	1 (0 to 5)	6 (0 to 22)		
Index and comparator tests negative (I−/C−) (patients without influenza B infection)	950 (941 to 950)	800 (792 to 800)	9 studies 3965 samples	⊕ ⊕ ⊝ ⊝^a^ LOW
Index test positive, comparator negative (I + /C−) (patients incorrectly classified as having influenza B infection)	0 (0 to 9)	0 (0 to 8)		

*C* Comparator, *CrI* Credible intervalI, *I* Index test, *RPP* Respiratory pathogen panel

Patient or population: adults and children with symptoms of acute lower respiratory infection

Setting: worldwide

Index test: Luminex NxTAG RPP™

Comparator tests: other RPP

Predicted sensitivity: 0.98 (95% CrI: 0.88 to 1.00) | Predicted specificity: 1.00 (95% CrI: 0.99 to 1.00)

aDowngraded two levels for risk of bias: there is high or unclear risk of bias on the patient selection and flow and timing domains for all included studies. Six studies were planed after the test was performed on patients selected by physician (e.g. respiratory symptoms) with no clear inclusion and exclusion criteria, which is at high risk of introducing bias for evaluating diagnostic test accuracy

Although seven studies provided data for influenza subtypes (Additional file 1: Table S13), we did not perform subgroup meta-analysis at this level due to the scarcity of data.

## Discussion

In this systematic review and meta-analysis of ten studies, including results from 4329 patient samples, we found that Luminex NxTAG RPP™ had a predicted mean sensitivity and specificity of 99% and 100% for detecting RSV, 97% and 100% for influenza A, and 98% and 100% for influenza B. If Luminex NxTAG RPP™ were used in a hypothetical population of 1000 persons with acute lower respiratory symptoms where 50 actually were infected with RSV-A or RSV-B (pre-test probability of 5%), we estimated that the test would correctly detect RSV in 50 people (50 I + /C + , 95% CrI 48–50), would not miss any infection (0 I−/C + , 95% CrI 0–2), and would not detect RSV in people in discordance with the comparator tests (0 I + /C-,−95% CrI 0–19) (Table 2). Similar results were seen with influenza A and B: with a pre-test probability of 5%, we would anticipate one I−/C + case and no I + /C− case (Tables 3 and 4).

However, these results must be treated with caution. We found a high risk of bias in most studies, particularly as regards patient selection, and a lack of clarity in many studies as to sample flow and timing. In several studies Luminex NxTAG RPP™ was performed on stored frozen respiratory samples with unclear storage duration. While the data generated by these studies is important for assay validation, it is more complex to generalise their results to other patient populations.

Respiratory pathogens including RSV and influenza viruses are often in the differential diagnosis for patients presenting with febrile illness. Consequently, RPP may be used clinically for diagnostic testing in undifferentiated fever cases. We did not find any studies matching our specific initial inclusion criterion of febrile patients. It may well be that Luminex NxTAG RPP™ performs equally well in patients with undifferentiated fever, but wider evaluation with prospective recruitment and clear inclusion criteria (symptomatic with fever and/or respiratory symptoms) should be conducted.

The uncertainty in this review is compounded by the wide range of comparator tests used. Alternative reference tests to RT-PCR include culture (the classic gold standard but time consuming and laborious); direct fluorescent antibody testing (requiring technical expertise and potentially subjective); serology (in general too slow to be of acute clinical relevance); and rapid immunoassays such as lateral flow tests, which may lack sensitivity [39]. Furthermore, in-house RT-PCRs are all likely to be unique in the first place, with different probe combinations and thus varying sensitivity and specificity. The wide range of reference tests is not isolated to our review—a previous meta-analysis of multiplex PCRs for diagnosis of respiratory infections showed similar findings [13]. This apparent lack of a single ‘gold standard’ may be explained by a reliance on national regulatory bodies to rigorously assess commercial tests to ensure quality and performance (e.g. Food and Drug Administration approval in the United States, CE-IVD marking in Europe) as opposed to large scale clinical studies evaluating each test against a ‘gold standard’. Indeed, under changing IVD regulation in Europe, laboratories are likely to need to justify the use of in-house tests over and above those that are commercially available. This lack of a gold standard might appear concerning, but with regulatory bodies ensuring baseline performance conformity, the broad range of test kits available means laboratories have the freedom to choose test kits that fit best with local demographics and individual laboratory logistics. What then becomes most important is ongoing quality assurance, in particular external quality assurance such as inter-laboratory exchange schemes.

In terms of the limitations of our review, we set out to review the diagnostic accuracy of Luminex NxTAG RPP™ for detecting RSV, influenza A and influenza B in febrile patients, to match FIEBRE study objectives [17], but we found no studies including participants enrolled on the basis of fever. We therefore expanded our review to include any study where clinical samples were evaluated with both Luminex NxTAG RPP™ and another assay, with obvious consequences in the applicability of our findings to patients with the common syndrome of febrile illness. Strengths of this review include a comprehensive literature search and a robust methodology with independent duplicate review and adherence to QUADAS-2 and GRADE methodology, and PRISMA guidelines. Furthermore, by using an extension to the HSROC model we have not assumed that any one test is a gold standard, but that all tests are imperfect measures of an underlying not directly observable (true disease) status or class [23]. This statistical method lends itself well to analysing the multiple comparator tests used in studies identified for this review and the inherent heterogeneity this brings as well as mitigating against the lack of a true gold standard reference test in this context.

## Conclusion

We found excellent sensitivity and specificity for the Luminex NxTAG RPP™ assay for RSV and influenza A and B, but within studies that were either limited to patients with respiratory symptoms, or with an unclear participant enrolment strategy. Further research is merited to ascertain whether Luminex NxTAG RPP™ will perform equally well among patients with febrile illness.

## Supplementary Information

**Additional file 1: Annex S1. **Search methodology. **Annex S2.** Databases. **Annex S3.** Statistical model. **Annex S4. **Literature search results. **Additional tables and figures.**

## Data Availability

Code used is publicly available at: https://github.com/shk313/diagnostic-test-metaanalysis/tree/main/RSV_Influenza. Data included in meta-analyses can be found in Additional file 1: Tables S11 and S12.

## Abbreviations

CrI: Credible interval
FIEBRE: Febrile Illness Evaluation in a Broad Range of Endemicities
HSROC: Hierarchical summary receiver operating characteristic
IVD: In vitro diagnostic
QUADAS-2: Quality assessment tool for diagnostic accuracy studies
RPPs: Respiratory pathogen panels
RSV: Respiratory syncytial virus
RT-PCR: Real-time polymerase chain reaction
