# Supplementary material for: Diagnostic accuracy of multiplex respiratory pathogen panels for influenza or respiratory syncytial virus infections: systematic review and meta-analysis
Source: BMC Infect Dis. 2022 Oct 13;22:785. doi: 10.1186/s12879-022-07766-9 (PMC9563105; doi:10.1186/s12879-022-07766-9)
Supplement: Supplementary file 1 — Additional file 1: Annex S1. Search methodology. Annex S2. Databases. Annex S3. Statistical model. Annex S4. Literature search results. Additional tables and figures. [file 12879_2022_7766_MOESM1_ESM.docx]

**Additional file 1**

**Diagnostic accuracy of multiplex respiratory pathogen panels for influenza or respiratory syncytial virus infections: systematic review and meta-analysis**

**Content**

[Annex 1. Search methodology 3](#_Toc91157955)

[Annex 2. Databases 10](#_Toc91157956)

[Annex 3. Statistical model 11](#_Toc91157957)

[Annex 4. Literature search results 15](#_Toc91157958)

[Supplementary tables 16](#_Toc91157959)

[Supplementary figures 39](#_Toc91157960)

# Annex S1. Search methodology

As recommended in the Cochrane Handbook for Systematic Reviews of Diagnostic Test Accuracy, a specific filter to identify diagnostic test accuracy studies was not included.

The tables below provide full details of all search strings used for bibliographic databases, with dates and number of references returned and notes explaining any unusual search techniques or syntax. The EndNote X9 import order is provided, as the deduplication technique keeps the first uploaded copy of the reference by default.

In all searches, numbers in parentheses at the end of each row show the number of hits retrieved.

**OvidSP Medline**

| Database name | Medline ALL |
| --- | --- |
| Database platform | OvidSP |
| Dates of database coverage | 1946 to September 21, 2020 |
| Date searched | 22 September 2020 |
| Searched by | JF |
| Number of results | 241 |
| EndNote import order | 1 |
| Number of results once duplicates removed | 239 |
| Search strategy notes | Search lines ending in a ‘/’ are subject heading searches. Search lines beginning ‘exp’ are exploded subject heading searches. Two-letter codes at the end of search lines designate the fields to search. Fields codes used are: TI: title AB: abstract KF: author keywords or/*x-y* combines search sets in the range *x-y* with Boolean operator OR. * is used for truncation of words. |

1. Respiratory Tract Infections/ (38694)
2. respiratory tract infection*.ti,ab,kf. (23631)
3. (arti or artis).ti,ab,kf. (829)
4. Respiratory Syncytial Virus Infections/ (6997)
5. respiratory syncytial viruses/ (5953
6. respiratory syncytial virus, human/ (2607)
7. respiratory syncytial.ti,ab,kf. (13994)
8. rsv.ti,ab,kf. (12210)
9. Influenza, Human/ (49651)
10. exp influenzavirus a/ (44202)
11. exp influenzavirus b/ (4349)
12. influenza.ti,ab,kf. (98459)
13. flu.ti,ab,kf. (13173)
14. influenzavirus.ti,ab,kf. (142)
15. grippe.ti,ab,kf. (338)
16. or/1-15 (174531)
17. High-Throughput Screening Assays/ (12221)
18. nxtag.ti,ab,kf. (17)
19. xtag.ti,ab,kf. (186)
20. respiratory pathogen panel*.ti,ab,kf. (34)
21. respiratory viral panel*.ti,ab,kf. (117)
22. respiratory panel*.ti,ab,kf. (139)
23. rpp.ti,ab,kf. (1661)
24. or/17-23 (14274)
25. 16 and 24 (399)
26. limit 25 to yr="2015 -Current" (241)

**OvidSP Embase**

| Database name | Embase Classic+Embase |
| --- | --- |
| Database platform | OvidSP |
| Dates of database coverage | 1947 to 2020 September 21 |
| Date searched | 22 September 2020 |
| Searched by | JF |
| Number of results | 494 |
| EndNote import order | 2 |
| Number of results once duplicates removed | 309 |
| Search strategy notes | Search lines ending in a ‘/’ are subject heading searches. Search lines beginning ‘exp’ are exploded subject heading searches. Two-letter codes at the end of search lines designate the fields to search. Fields codes used are: TI: title AB: abstract KW: author keywords or/*x-y* combines search sets in the range *x-y* with Boolean operator OR. * is used for truncation of words. |

1. respiratory tract infection/ (64807)
2. viral respiratory tract infection/ (4290)
3. (arti or artis).ti,ab,kw. (1350)
4. respiratory syncytial virus infection/ (5375)
5. exp human respiratory syncytial virus/ (4973)
6. respiratory syncytial.ti,ab,kw. (17800)
7. rsv.ti,ab,kw. (16854)
8. exp influenza/ (96964)
9. influenza virus/ (23101)
10. exp influenzavirus a/ (13284)
11. exp influenzavirus b/ (1523)
12. influenza.ti,ab,kw. (121205)
13. flu.ti,ab,kw. (21026)
14. influenzavirus.ti,ab,kw. (219)
15. grippe.ti,ab,kw. (270)
16. or/1-15 (241753)
17. high throughput screening/ (36332)
18. nxtag.ti,ab,kw. (30)
19. xtag.ti,ab,kw. (319)
20. respiratory pathogen panel*.ti,ab,kw. (76)
21. respiratory viral panel*.ti,ab,kw. (334)
22. respiratory panel*.ti,ab,kw. (296)
23. rpp.ti,ab,kw. (2314)
24. or/17-23 (39539)
25. 16 and 24 (847)
26. limit 25 to yr="2015 -Current" (494)

**OvidSP Global Health**

| Database name | Global Health |
| --- | --- |
| Database platform | OvidSP |
| Dates of database coverage | 1910 to 2020 week 37 |
| Date searched | 22 September 2020 |
| Searched by | JF |
| Number of results | 98 |
| EndNote import order | 3 |
| Number of results once duplicates removed | 8 |
| Search strategy notes | Search lines ending in a ‘/’ are subject heading searches. Search lines beginning ‘exp’ are exploded subject heading searches. Two-letter codes at the end of search lines designate the fields to search. Fields codes used are: TI: title AB: abstract or/*x-y* combines search sets in the range *x-y* with Boolean operator OR. * is used for truncation of words. |

1. respiratory diseases/ (71164)
2. respiratory tract infection*.ti,ab. (10188)
3. (arti or artis).ti,ab. (292)
4. human respiratory syncytial virus/ (6110)
5. respiratory syncytial.ti,ab. (6613)
6. rsv.ti,ab. (4690)
7. exp influenza/ (40457)
8. influenza viruses/ (22922)
9. influenzavirus a/ (20232)
10. influenzavirus b/ (3448)
11. influenza.ti,ab. (45421)
12. flu.ti,ab. (4566)
13. influenzavirus.ti,ab. (453)
14. grippe.ti,ab. (134)
15. or/1-14 (119235)
16. nxtag.ti,ab. (12)
17. xtag.ti,ab. (104)
18. respiratory pathogen panel*.ti,ab. (23)
19. respiratory viral panel*.ti,ab. (85)
20. respiratory panel*.ti,ab. (91)
21. rpp.ti,ab. (171)
22. or/16-21 (406)
23. 15 and 22 (186)
24. limit 23 to yr="2015 -Current" (98)

**Wiley Cochrane CENTRAL database**

| Database name | Cochrane Central Register of Controlled Trials |
| --- | --- |
| Database platform | Wiley |
| Dates of database coverage | Issue 9 of 12, September 2020 |
| Date searched | 22 September 2020 |
| Searched by | JF |
| Number of results | 19 |
| EndNote import order | 7 |
| Number of results once duplicates removed | 10 |
| Search strategy notes | * is used for truncation.  Searches ending :ti,ab,kw search the title, abstract and keywords.  Note, numbers in parentheses are results across all Cochrane databases. |

#1 MeSH descriptor: [Respiratory Tract Infections] this term only (2251)

#2 ("respiratory tract infection*"):ti,ab,kw (6767)

#3 (arti or artis):ti,ab,kw (91)

#4 MeSH descriptor: [Respiratory Syncytial Virus Infections] this term only (307)

#5 MeSH descriptor: [Respiratory Syncytial Viruses] this term only (102)

#6 MeSH descriptor: [Respiratory Syncytial Virus, Human] this term only (58)

#7 ("respiratory syncytial"):ti,ab,kw (921)

#8 (rsv):ti,ab,kw (819)

#9 MeSH descriptor: [Influenza, Human] this term only (2652)

#10 MeSH descriptor: [Influenzavirus A] explode all trees (861)

#11 MeSH descriptor: [Influenzavirus B] explode all trees (283)

#12 (influenza):ti,ab,kw (7330)

#13 (flu):ti,ab,kw (2898)

#14 (influenzavirus):ti,ab,kw (23)

#15 (grippe):ti,ab,kw (45)

#16 #1 or #2 or #3 or #4 or #5 or #6 or #7 or #8 or #9 or #10 or #11 or #12 or #13 or #14 or #15 (17553)

#17 MeSH descriptor: [High-Throughput Screening Assays] this term only (8)

#18 (nxtag):ti,ab,kw (3)

#19 (xtag):ti,ab,kw (6)

#20 ("respiratory pathogen panel*"):ti,ab,kw (4)

#21 ("respiratory viral panel*"):ti,ab,kw (3)

#22 ("respiratory panel*"):ti,ab,kw (15)

#23 (rpp):ti,ab,kw (360)

#24 #17 or #18 or #19 or #20 or #21 or #22 or #23 (393)

#25 #16 and #24 (19)

**Clarivate Analytics Web of Science**

| Database name | Science Citation Index Expanded Social Sciences Citation Index |
| --- | --- |
| Database platform | Clarivate Analytics Web of Science |
| Dates of database coverage | Both databases 1970-present. Data last updated 2020-09-21 |
| Date searched | 22 September 2020 |
| Searched by | JF |
| Number of results | 141 |
| EndNote import order | 5 |
| Number of results once duplicates removed | 7 |
| Search strategy notes | * is used for truncation.  All searches run across Indexes=SCI-EXPANDED, SSCI Timespan=All years |

#1 TS=("respiratory tract infection*" or arti or artis or "respiratory syncytial" or rsv or influenza or flu or influenzavirus or grippe) (45,863)

#2 TS=(nxtag or xtag or "respiratory pathogen panel*" or "respiratory viral panel*" or "respiratory panel*" or rpp) (849)

#3 #2 AND #1 (141)

**Ebsco Africa-Wide Information**

| Database name | Africa-Wide Information |
| --- | --- |
| Database platform | Ebsco |
| Dates of database coverage | Complete database as of search date |
| Date searched | 22 September 2020 |
| Searched by | JF |
| Number of results | 4 |
| EndNote import order | 4 |
| Number of results once duplicates removed | 0 |
| Search strategy notes | Two-letter codes at the beginning of search lines designate the fields to search. Fields codes used are: TI: title AB: abstract KW: keywords * is used for truncation of words. |

S1 (TI ("respiratory tract infection*" or arti or artis or "respiratory syncytial" or rsv or influenza or flu or influenzavirus or grippe)) or (AB ("respiratory tract infection*" or arti or artis or "respiratory syncytial" or rsv or influenza or flu or influenzavirus or grippe)) or (KW ("respiratory tract infection*" or arti or artis or "respiratory syncytial" or rsv or influenza or flu or influenzavirus or grippe)) (12,551)

S2 (TI (nxtag or xtag or "respiratory pathogen panel*" or "respiratory viral panel*" or "respiratory panel*" or rpp)) or (AB (nxtag or xtag or "respiratory pathogen panel*" or "respiratory viral panel*" or "respiratory panel*" or rpp)) or (KW (nxtag or xtag or "respiratory pathogen panel*" or "respiratory viral panel*" or "respiratory panel*" or rpp)) (126)

S3 S1 AND S2 (5)

S4 S3 Limiters - Year Published: 2015-2020 (4)

**Scopus**

| Database name | Scopus |
| --- | --- |
| Database platform | Scopus.com |
| Dates of database coverage | Complete database as of search date |
| Date searched | 22 September 2020 |
| Searched by | JF |
| Number of results | 210 |
| EndNote import order | 6 |
| Number of results once duplicates removed | 26 |
| Search strategy notes | * is used for truncation of words. |

( TITLE-ABS-KEY ( "respiratory tract infection*" OR arti OR artis OR "respiratory syncytial" OR rsv OR influenza OR flu OR influenzavirus OR grippe ) ) AND ( TITLE-ABS-KEY ( nxtag OR xtag OR "respiratory pathogen panel*" OR "respiratory viral panel*" OR "respiratory panel*" OR rpp ) ) AND ( LIMIT-TO ( PUBYEAR , 2020 ) OR LIMIT-TO ( PUBYEAR , 2019 ) OR LIMIT-TO ( PUBYEAR , 2018 ) OR LIMIT-TO ( PUBYEAR , 2017 ) OR LIMIT-TO ( PUBYEAR , 2016 ) OR LIMIT-TO ( PUBYEAR , 2015 ) )

**LILACS**

| Database name | LILACS |
| --- | --- |
| Database platform | WHO Global Index Medicus |
| Dates of database coverage | Complete database as of search date |
| Date searched | 22 September 2020 |
| Searched by | JF |
| Number of results | 2 |
| EndNote import order | 8 |
| Number of results once duplicates removed | 1 |
| Search strategy notes | None |

(tw:("respiratory tract" or arti or artis or "respiratory syncytial" or rsv or influenza or flu or influenzavirus or grippe)) AND (tw:(nxtag or xtag or "respiratory pathogen panel" or "respiratory pathogen panels" or "respiratory viral panel" or "respiratory viral panels" or "respiratory panel" or "respiratory panels" or rpp))

**Global Index Medicus**

| Database name | Global Index Medicus |
| --- | --- |
| Database platform | WHO Global Index Medicus |
| Dates of database coverage | Complete database as of search date |
| Date searched | 22 September 2020 |
| Searched by | JF |
| Number of results | 14 |
| EndNote import order | 9 |
| Number of results once duplicates removed | 9 |
| Search strategy notes | None |

(tw:("respiratory tract" or arti or artis or "respiratory syncytial" or rsv or influenza or flu or influenzavirus or grippe)) AND (tw:(nxtag or xtag or "respiratory pathogen panel" or "respiratory pathogen panels" or "respiratory viral panel" or "respiratory viral panels" or "respiratory panel" or "respiratory panels" or rpp))

# Annex S2. Databases

The following bibliographic databases were searched on 22 September 2020.

- OvidSP Medline ALL, 1946 to September 21, 2020.
- OvidSP Embase, 1947 to 2020 September 21.
- OvidSP Global Health, 1910 to 2020 week 37.
- Wiley Cochrane Central Register of Controlled Trials, Issue 9 of 12, September 2020.
- Clarivate Analytics Web of Science, Data last updated 2020-09-21:
  - Science Citation Index-Expanded, 1970-present;
  - Social Sciences Citation Index, 1970-present.
- Elsevier Scopus, complete database.
- Ebsco Africa-Wide Information, complete database.
- WHO LILACS, complete database.
- WHO Global Index Medicus, complete database.

# Annex S3. Statistical model

The complete model to estimate the sensitivity and specificity of the index test in each study, and hence predict sensitivity and specificity in a future study, consists of two hierarchical levels. The first level captures between-study variations in the sensitivity and specificity through a Hierarchical Summary Receiver Operating Characteristic (HSROC) model, which assumes that sensitivity and specificity across studies lie on an ROC curve. The second level accounts for the unknown true disease status of all participants and potential conditional dependence between diagnostic tests through the use of a random-effect latent class model.

Starting with the latent class model, assume we have a sample of N individuals who have all undergone two different dichotomous tests defined by $T_{r} (r =1,2), and let t_{ri}$ be a random variable denoting the outcome from test $r$for individual $i, i=1,..,N$. A positive test result for an individualis denoted by $t_{ri}=1$, and a negative test result by $t_{ri}=0.$ Also assume that the unknown true disease status of an individual, denoted $D$, can take one of two values: ‘diseased’ ($D=1)$ or ‘non-diseased’ ($D=0)$.The true (latent) disease status of the $i$^th^ individual is denoted $d_{i} .$ Assuming conditional independence between the two tests, we can write the joint distribution as:

| $\Pr\left( T_{1}= t_{1},T_{2}=t_{2} \right)=$  $Pr(T_{1}\vert D=1)Pr(T_{2}\vert D=1)Pr(D=1)+Pr(T_{1}\vert D=0)Pr(T_{2}\vert D=0)Pr(D=0)$ | 1 |
| --- | --- |

For the *r^t^*^h^ test, the sensitivity $(Se)$ and specificity $(Sp)$ can be written as $\mathrm{Se}_{r}=Pr(T_{r} =1|D=1)$ and$\mathrm{Sp}_{r}=Pr(T_{r}=0|D=0)$ respectively. We can also define prevalence as $\pi=Pr(D=1)$. We can then specify equation 1 in terms of sensitivity, specificity and prevalence:

| $\Pr\left( T_{1}= t_{1},T_{2}=t_{2} \right)=$  $\mathrm{Se}_{1}\mathrm{Se}_{2}(1-\mathrm{Se}_{1})(1-\mathrm{Se}_{2})\pi+(1-\mathrm{Sp}_{1})(1-\mathrm{Sp}_{2})\mathrm{Sp}_{1}\mathrm{Sp}_{2}(1-\pi)$ | 2 |
| --- | --- |

Following this, we can then define the likelihood for this latent class model assuming conditional independence as:

| $L = \prod_{i=1}^{N} (\pi{\mathrm{Se}_{1}}^{t_{1i}}{\mathrm{Se}_{2}}^{t_{2i}}(1-{\mathrm{Se}_{1})}^{1-t_{1i}}(1-{\mathrm{Se}_{2})}^{1-t_{2i}})+((1-\pi){\mathrm{Sp}_{1}}^{1-t_{1i}}{\mathrm{Sp}_{2}}^{1-t_{2i}}(1-{\mathrm{Sp}_{1})}^{t_{1i}}(1-{\mathrm{Sp}_{2})}^{t_{2i}})$ | 3 |
| --- | --- |

We account for conditional dependence between tests where necessary by allowing the sensitivity from each test to depend on an individual level random effect $s_{i}$. We assume that the sensitivity in the conditionally dependent model takes the form (1):

| ${Pr(T_{\mathrm{ri}}= 1\vert D_{i}=1,) =Se}_{\mathrm{ri}} = g^{-1}(a_{rd=1} + b_{rd=1}s_{i})$ | 4 |
| --- | --- |

where $g\left( \cdot\right)$ is a link function. In this study we use the logit link, $g^{-1}(y) = 1/(1+e^{-y})$, $a_{rd=1}$ and $b_{rd=1}$ are unknown parameters to be estimated where b describes the strength of dependence between two tests and the random effect $s_{i}$ follows a standard normal distribution. The subject-specific random-effect $s_{i}$ represents some unobserved characteristic for example infection intensity, that indirectly creates dependence between tests.

The study-level latent class model (equation 3 and 4) is then linked to the between-study level using a HSROC model (equation 5) which models between study variations by assuming that test sensitivity and specificity lie on an ROC curve. In particular, each study $j$ $(j=1,..,J)$provides the 2x2 table between the test of interest, hereafter called the index test, which is the same in all studies, and a comparator test which may differ between studies. We let $T_{1j}$ denote the index test outcomes in each study and we let $T_{2j}$ denote the comparator test outcomes in each study. In line with previous descriptions (2,3), we define the sensitivity and specificity of the index test in the $j$th study by:

| $\mathrm{logit}{(Se}_{1j})=Pr(T_{1j}=1\vert D=1) =-(\theta_{j} - \alpha_{j}/2)/exp(\beta/2)$  ${logit(Sp}_{1j})=Pr(T_{1j}=0\vert D=0) =(\theta_{j} + \alpha_{j}/2)/exp(-\beta/2)$ | 5 |
| --- | --- |

where $\theta_{j}$ represents the positivity criteria for study $j$. The positivity criteria, or cut-off value, models the dependence between the true positive fraction and false positive fraction in each study. $\alpha_{j}$ represents the diagnostic accuracy and measures the mean difference in test accuracy between individuals ‘diseased’ and individuals ‘non-diseased’ in study $j$. $\beta$ the scale parameter, allows differences in the variation of outcomes between disease positive and disease negative individuals thus allowing asymmetry in the ROC curve. Both $\theta$ and$\alpha$ parameters are modelled as random effects with independent normal distributions to incorporate variation between studies:

| $\theta_{j}\sim N(\Theta, \sigma_{\theta})$  $\alpha_{j} \sim N(\Lambda, \sigma_{\alpha})$ | 6 |
| --- | --- |

Equations 5 and 6 represent the model specification for the RSV model described in the main text, and it includes no covariates. However, for Influenza we also wanted to allow for heterogeneity between the Influenza types, influenza A and influenza B. For this we allow the means of $\alpha, \theta$ and $\beta$ to be a function of a study-level covariate. We let $Z_{\mathrm{Aj}}$take the value 1 if the data are for influenza A and 0 otherwise, and $Z_{\mathrm{Bj}}$take the value 1 if the data are for influenza B and 0 otherwise. We also allowed the standard deviation of $\alpha$ and $\theta$ to vary by Influenza type. The distribution of $\alpha$ and its standard deviation are then assumed to be:

| $\alpha_{j}\sim N(\Lambda_{A}Z_{\mathrm{Aj}}+\Lambda_{B}Z_{\mathrm{Bj}},\sigma_{\alpha})$  $\sigma_{\alpha}\sim N(0,1)$ | 7 |
| --- | --- |

where $\Delta_{type}$ represents the coefficient for each Influenza type. We also allowed the standard deviation of $\alpha$ and $\theta$ to vary by Influenza type.

We present pooled estimates of sensitivity and specificity which are given by:

| $\mathrm{logit}\left( \mathrm{Pooled}\mathrm{Se}_{1} \right)=-\left( \left( \Theta-\Lambda/2 \right)/\exp\left( \beta/2 \right) \right)$  $logit (Pooled \mathrm{Sp}_{1})=((\Theta+\Lambda/2)/exp(-\beta/2))$ | 8 |
| --- | --- |

And, where we included a covariate for Influenza type, $\Theta,\Lambda$ and $\beta$ are replaced by the corresponding coefficient. For example, to estimate the pooled sensitivity and specificity of Influenza A:

| $\mathrm{logit}\left( \mathrm{Pooled}{Influenza A Se}_{1} \right)=-\left( \left( \Theta_{A}-\Lambda_{A}/2 \right)/\exp\left( \beta_{A}/2 \right) \right)$  $logit (Pooled Influenza A \mathrm{Sp}_{1})=((\Theta_{A}+\Lambda_{A}/2)/exp(-\beta_{A}/2))$ | 9 |
| --- | --- |

Importantly, we also present a prediction of sensitivity and specificity in a new study. Predicted estimates are important because the pooled estimates in a meta-analysis only represent an average estimate among the studies included in the analysis. Predicted estimates on the other hand account for the variation captured through the modelling framework and can be used as priors for sensitivity and specificity of the index test in a new study. We predict sensitivity and specificity by replacing $\Theta$ with $\theta_{newstudy}$ and $\Lambda$ with $\alpha_{newstudy}$:

| $\theta_{newstudy}\sim N\left( \Theta,\sigma_{\theta} \right)$  $\alpha_{newstudy}\sim N\left( \Lambda,\sigma_{\alpha} \right)$ | 10 |
| --- | --- |

**Prior Specification**

Priors for both models are in line with those used by Dendukuri *et al.* (3), where the priors were chosen ensure a uniform distribution for the pooled estimate of sensitivity over 0.5 to 1. We use the following priors: $\Theta$~𝑁(0,1.5), $\Lambda$~𝑁(0,2) and, 𝛽~𝑈𝑛𝑖𝑓𝑜𝑟𝑚(−0.75,0.75). Variance parameters $\sigma_{\theta}$ 𝑎𝑛𝑑 $\sigma_{\alpha}$ follow zero-truncated standard normal distributions. We allow prevalence, and sensitivity and specificity of the index test in each study to be uniform over 0 to 1. However we assume the reference test to be superior to the toss of a coin and is uniform over 0.5 to 1.

**Sensitivity Analysis**

Sensitivity analyses were conducted to assess the robustness of our results to choice of priors and to assumptions about conditional dependence between tests. For the final model presented, we allowed the variance parameters, $\sigma_{\theta}$ and $\sigma_{\alpha}$, to follow a 𝑔𝑎𝑚𝑚𝑎(0.5,0.5) prior distribution. The pooled and predicted estimates from these models did not differ greatly to those in the primary analyses.

**References:**

1. Dendukuri N, Joseph L. Bayesian approaches to modeling the conditional dependence between multiple diagnostic tests. Biometrics. 2001;57(1):158-67.
2. Rutter CM, Gatsonis CA. A hierarchical regression approach to meta-analysis of diagnostic test accuracy evaluations. Stat Med. 2001;20(19):2865-84.
3. Dendukuri N, Schiller I, Joseph L, Pai M. Bayesian meta-analysis of the accuracy of a test for tuberculous pleuritis in the absence of a gold standard reference. Biometrics. 2012;68(4):1285-93.

# Annex S4. Literature search results

A total of 1223 results were retrieved by the search. 614 (50%) were identified as duplicates. Number of results pre-and post-deduplication are listed in the table below. One additional reference was identified by contacting experts and Luminex manufacturers to make a total of 610.

| **Database name** | **Total number of results** | **Number of results once duplicates removed** |
| --- | --- | --- |
| Medline | 241 | 239 |
| Embase | 494 | 309 |
| Global Health | 98 | 8 |
| Africa-Wide Information | 4 | 0 |
| Cochrane Central Register of Controlled Trials | 19 | 10 |
| Global Index Medicus | 14 | 9 |
| Web of Science databases (both searched together) | 141 | 7 |
| Scopus | 210 | 26 |
| LILACS | 2 | 1 |
| Expert/Manufacturer Consultation | 1 | 1 |
| **Total** | **1223** | **610** |

# Additional file 1: Tables

**Table S1A. Beckmann 2016: study characteristics**

| **Participant sampling** | Cross sectional study with:  (A) For 115 samples, retrospective data collection for the index test results  (B) For 167 samples, prospective collection of consecutive samples for index and reference testing | |
| --- | --- | --- |
| **Participant characteristics and setting** | **Country** | Switzerland |
|  | **Study centres** | Data collection from the University Hospital in Basel for adults and the University Children’s Hospital in Basel for children |
|  | **Study dates** | (A) September to December 2014  (B) March to April 2015 |
|  | **Inclusion/exclusion criteria** | Not reported |
|  | **Sample size** | 282 samples from 271 patients |
|  | **Age** | Adults and children  Median age 42 years, interquartile range 2 to 63 years |
|  | **Sex** | 119 (43.9%) females |
|  | **Respiratory tract infection symptoms** | Not reported |
|  | **Febrile patients** | Not reported |
|  | **Inpatient/outpatient** | Not reported |
|  | **Samples collected** | 199 (71%) nasopharyngeal swabs  76 (27%) broncho-alveolar lavages  4 throat swabs  2 tracheal secretions  1 sputum |
| **Index test** | NxTAG-RPP (Luminex, MV’s- Hertogenbosch, The Netherlands) | |
| **Comparator tests** | **1^st^ comparator test** | Multiplex-ligation-NAT based RespiFinder-221 (RF-22)  RespiFinder-221 (RF-22, PathoFinder, Maastricht, The Netherlands). This platform detects 22 different pathogens using a multiplex ligation-dependent probe amplification (MLPA) coupled to capillary electrophoresis.  Failure of the RF-22 with invalid internal controls or other technical issues were seen in less than 2%, and required repeat testing. |
|  | **2^nd^ comparator test** | For discordant results only: in-house quantitative PCRs (QNAT) |
| **Flow and timing** | **Duration of sample storage** | (A) <8 months for 115 stored samples, according to study date  (B) Parallel prospective analysis for 167 consecutive samples |
|  | **Intervention and comparator tests performed in same sample** | Yes |
|  | **Missing data** | None |
| **Notes** | **Source of funding** | Luminex Molecular Diagnostics provided the NxTAG-RPP reagents and MagPIX instrument |
|  | **Declared conflict of interests** | Authors declared no conflict of interest |

**Table S1B. Beckmann 2016: methodological quality (QUADAS-2)**

| **Item** | **Authors’ judgment** | **Risk of bias** | **Applicability concerns** |
| --- | --- | --- | --- |
| **Domain 1: Patient selection**  Risk of bias: Could the selection of patients have introduced bias?  Applicability: Are there concerns that the included patients and setting do not match the review question? | | | |
| Was a consecutive or random sample of patients enrolled? | 115 no  167 consecutive |  |  |
| Was a case-control design avoided? | Yes |  |  |
| Did the study avoid inappropriate exclusions? | Unclear |  |  |
| Judgment on risk of bias and applicability concerns |  | High | Unclear (unclear characteristics of participants) |
| **Domain 2: Index test**  Risk of Bias: Could the conduct or interpretation of the index test have introduced bias?  Applicability: Are there concerns that the index test, its conduct, or interpretation differ from the review question? | | | |
| Were the index test results interpreted without knowledge of the results of the reference standard? | 115 no  167 unclear |  |  |
| If a threshold was used, was it pre-specified?  (always yes for NxTAG) | Yes |  |  |
| Judgment on risk of bias and applicability concerns |  | Low | Low |
| **Domain 3: Comparator test**  Risk of Bias: Could the comparator test, its conduct, or its interpretation have introduced bias?  Applicability: Are there concerns that the target condition as defined by the comparator test does not match the question? | | | |
| Is the comparator test likely to correctly classify the target condition? | Yes |  |  |
| Were the comparator test results interpreted without knowledge of the results of the index tests? | 115 yes  167 unclear |  |  |
| Judgment on risk of bias and applicability concerns |  | Low (unblinding unlikely to introduce interpretation bias) | Low |
| **Domain 4: Flow and timing**  Risk of Bias: Could the patient flow have introduced bias? | | | |
| Was there an appropriate interval between index test and comparator test? | Unclear |  |  |
| Did all patients receive a comparator test? | Yes |  |  |
| Did all patients receive the same comparator test? | Yes |  |  |
| Were all patients included in the analysis? | Yes |  |  |
| Judgment on risk of bias and applicability concerns |  | Unclear | Not applicable |

**Table S2A. Brotons 2016: study characteristics**

| **Participant sampling** | Cross-sectional study with prospective recruitment of consecutive children | |
| --- | --- | --- |
| **Participant characteristics and setting** | **Country** | Spain |
|  | **Study centres** | Hospital Sant Joan de Déu (HSJD), Barcelona |
|  | **Study dates** | June to November 2015, out of the influenza season |
|  | **Inclusion/exclusion criteria** | Children and adolescents <18 years with nonspecific symptoms of acute low respiratory infection admitted to HSJD during the study period.  Acute low respiratory infection was defined as the presence of at least one specific lower respiratory tract sign (fast or difficulty breathing, chest wall indrawing) and/or abnormal auscultatory findings (crackles/crepitations or bronchial breath sounds). |
|  | **Sample size** | 319 samples included in the analysis  (320 samples collected but 1 sample with invalid result with NxTAG) |
|  | **Age** | Not reported |
|  | **Sex** | Not reported |
|  | **Respiratory tract infection symptoms** | Part of the inclusion criteria |
|  | **Febrile patients** | Not reported |
|  | **Inpatient/outpatient** | Inpatients |
|  | **Samples collected** | Nasopharyngeal aspirates |
| **Index test** | NxTAG-RPP RUO (Luminex Molecular Diagnostics, Toronto, Ontario, Canada) | |
| **Comparator tests** | **1^st^ comparator test** | Multiplex Anyplex II RV16 assay (Seegene, Inc., Seoul, South Korea).  This quantitative multiplex PCR detects 16 targets and requires a separate cDNA synthesis step before performing the multiplex PCR step. |
|  | **2^nd^ comparator test** | For discordant results only: bidirectional sequencing.  PCR and Sanger sequencing primers were designed not to overlap with the primers used in the NxTAG RPP RUO panel. |
| **Flow and timing** | **Duration of sample storage** | Collected in phosphate-buffered saline according to standard operational procedures of the hospital’s clinical laboratory.  When we collected repeated samples from the same patient, only the first sample was considered for evaluation. Specimens were aliquoted into two parts, frozen at 80°C, and thawed prior to testing. Sample batches were run two to three times a week to optimize the use of reagents. |
|  | **Intervention and comparator tests performed in same sample** | Yes, the first aliquot of each sample was processed by Anyplex II RV16 and the second aliquot was tested by NxTAG RPP |
|  | **Missing data** | None |
| **Notes** | **Source of funding** | Luminex Corporation, Fondo Europeo de Desarrollo Regional (FEDER) and the Ministry of Science and Innovation, Institute of Health Carlos III. |
|  | **Declared conflict of interests** | The authors declared that the funders had no role in the study design, data collection and interpretation, or the decision to submit the work for publication. |

**Table S2B. Brotons 2016: methodological quality (QUADAS-2)**

| **Item** | **Authors’ judgement** | **Risk of bias** | **Applicability concerns** |
| --- | --- | --- | --- |
| **Domain 1: Patient selection**  Risk of bias: Could the selection of patients have introduced bias?  Applicability: Are there concerns that the included patients and setting do not match the review question? | | | |
| Was a consecutive or random sample of patients enrolled? | Yes |  |  |
| Was a case-control design avoided? | Yes |  |  |
| Did the study avoid inappropriate exclusions? | Yes |  |  |
| Judgment on risk of bias and applicability concerns |  | Low | Low |
| **Domain 2: Index test**  Risk of Bias: Could the conduct or interpretation of the index test have introduced bias?  Applicability: Are there concerns that the index test, its conduct, or interpretation differ from the review question? | | | |
| Were the index test results interpreted without knowledge of the results of the reference standard? | Unclear |  |  |
| If a threshold was used, was it pre-specified?  (always yes for NxTAG) | Yes |  |  |
| Judgment on risk of bias and applicability concerns |  | Low | Low |
| **Domain 3: Comparator test**  Risk of Bias: Could the comparator test, its conduct, or its interpretation have introduced bias?  Applicability: Are there concerns that the target condition as defined by the comparator test does not match the question? | | | |
| Is the comparator test likely to correctly classify the target condition? | Yes (PCR confirms discordant results) |  |  |
| Were the comparator test results interpreted without knowledge of the results of the index tests? | Unclear |  |  |
| Judgment on risk of bias and applicability concerns |  | Low (unclear blinding unlikely to introduce bias) | Low |
| **Domain 4: Flow and timing**  Risk of Bias: Could the patient flow have introduced bias? | | | |
| Was there an appropriate interval between index test and comparator test? | Yes |  |  |
| Did all patients receive a comparator test? | Yes |  |  |
| Did all patients receive the same comparator test? | Yes for the reference test 1 |  |  |
| Were all patients included in the analysis? | Yes (1 excluded with reasons) |  |  |
| Judgment on risk of bias and applicability concerns |  | Low | Not applicable |

**Table S3A. Chan 2017: study characteristics**

| **Participant sampling** | Retrospective analysis of collected samples | |
| --- | --- | --- |
| **Participant characteristics and setting** | **Country** | Hong Kong, China |
|  | **Study centres** | Clinical sample collection from the Queen Mary Hospital in Hong Kong , and RT-PCR performed at the Hong Kong Government Virus Laboratory |
|  | **Study dates** | Not reported |
|  | **Inclusion/exclusion criteria** | Patients with respiratory tract infections and with clinical PCR results. |
|  | **Sample size** | 133 samples |
|  | **Age** | Adults and children  Mean age 32.5 years, range 2 months to 99 years |
|  | **Sex** | 58 (43.6%) females |
|  | **Respiratory tract infection symptoms** | Part of the inclusion criteria |
|  | **Febrile patients** | Not reported |
|  | **Inpatient/outpatient** | Not reported |
|  | **Samples collected** | Nasopharyngeal aspirates |
| **Index test** | NxTAG-RPP, CE-IVD (Luminex Molecular Diagnostics, Toronto, Canada) | |
| **Comparator tests** | **1^st^ comparator test** | Direct immunofluorescence (D3 Ultra 8 DFA Respiratory Virus Screening and Identification Kit, Diagnostic Hybrids, Inc. (Quidel), USA) + RT-PCR |
|  | **2^nd^ comparator test** | Some samples are also tested with Luminex xTAG Respiratory Viral Panel Fast v2 (Luminex Molecular Diagnostics, Toronto, Canada) and with FilmArray Respiratory Panel (RP) (bioMérieux, Marcy l'´Etoile, France), but no data were available to fill 2x2 tables for comparison with NxTAG RPP. |
| **Flow and timing** | **Duration of sample storage** | Not reported |
|  | **Intervention and comparator tests performed in same sample** | Yes |
|  | **Missing data** | None |
| **Notes** | **Source of funding** | This work was partly supported by the donations of Larry Chi-Kin Yung and Hui Hoy and Chow Sin Lan Charity Fund Limited  The authors also obtained funding from the Consultancy Service for Enhancing Laboratory Surveillance of Emerging Infectious Diseases, the Department of Health, Hong Kong Special Administrative Region; the University Development Fund and the Committee for Research and Conference Grant; the University of Hong Kong; the Collaborative Innovation Center for Diagnosis and Treatment of Infectious Diseases, the Ministry of Education of China; and the Commissioned Research on Control of Infectious Diseases (Phase III) of the Health and Medical Research Fund (HKM-15-M-04) of the Food and Health Bureau of the HKSAR Government. |
|  | **Declared conflict of interests** | Authors declared no conflict of interest |

**Table S3B. Chan 2017: methodological quality (QUADAS-2)**

| **Item** | **Authors’ judgement** | **Risk of bias** | **Applicability concerns** |
| --- | --- | --- | --- |
| **Domain 1: Patient selection**  Risk of bias: Could the selection of patients have introduced bias?  Applicability: Are there concerns that the included patients and setting do not match the review question? | | | |
| Was a consecutive or random sample of patients enrolled? | No |  |  |
| Was a case-control design avoided? | Yes |  |  |
| Did the study avoid inappropriate exclusions? | Unclear |  |  |
| Judgment on risk of bias and applicability concerns |  | High | Low |
| **Domain 2: Index test**  Risk of Bias: Could the conduct or interpretation of the index test have introduced bias?  Applicability: Are there concerns that the index test, its conduct, or interpretation differ from the review question? | | | |
| Were the index test results interpreted without knowledge of the results of the reference standard? | Unclear |  |  |
| If a threshold was used, was it pre-specified?  (always yes for NxTAG) | Yes |  |  |
| Judgment on risk of bias and applicability concerns |  | Low | Low |
| **Domain 3: Comparator test**  Risk of Bias: Could the comparator test, its conduct, or its interpretation have introduced bias?  Applicability: Are there concerns that the target condition as defined by the comparator test does not match the question? | | | |
| Is the comparator test likely to correctly classify the target condition? | Yes |  |  |
| Were the comparator test results interpreted without knowledge of the results of the index tests? | Yes |  |  |
| Judgment on risk of bias and applicability concerns |  | Low | Low |
| **Domain 4: Flow and timing**  Risk of Bias: Could the patient flow have introduced bias? | | | |
| Was there an appropriate interval between index test and comparator test? | Unclear |  |  |
| Did all patients receive a comparator test? | Yes |  |  |
| Did all patients receive the same comparator test? | Yes |  |  |
| Were all patients included in the analysis? | Yes |  |  |
| Judgment on risk of bias and applicability concerns |  | Unclear | Not applicable |

**Table S4A. Chen 2016: study characteristics**

| **Participant sampling** | Cross sectional study with prospective data collection | |
| --- | --- | --- |
| **Participant characteristics and setting** | **Country** | Hong Kong, China |
|  | **Study centres** | University of Hong Kong/Hospital Authority Hong Kong West Hospital Cluster |
|  | **Study dates** | 1 January to 31 December 2015 |
|  | **Inclusion/exclusion criteria** | Patients with symptomatic respiratory tract infections |
|  | **Sample size** | 284 samples from 277 patients |
|  | **Age** | Not reported |
|  | **Sex** | Not reported |
|  | **Respiratory tract infection symptoms** | Part of the inclusion criteria |
|  | **Febrile patients** | Not reported |
|  | **Inpatient/outpatient** | Not reported |
|  | **Samples collected** | Nasopharyngeal swabs |
| **Index test** | NxTAG-RPP (Luminex Molecular Diagnostics, Toronto, Canada) | |
| **Comparator tests** | **1^st^ comparator test** | BioFire FilmArray Respiratory Panel (bioMérieux, Marcy l’Etoile, France) |
|  | **2^nd^ comparator test** | None for the purpose of our review |
| **Flow and timing** | **Duration of sample storage** | Not reported, but probably <1 year based on time between sample collection and submission of their manuscript |
|  | **Intervention and comparator tests performed in same sample** | Yes |
|  | **Missing data** | None |
| **Notes** | **Source of funding** | The NxTAG-RPP reagents were provided by Luminex Molecular Diagnostics. |
|  | **Declared conflict of interests** | Authors declared no conflict of interest |

**Table S4B. Chen 2016: methodological quality (QUADAS-2)**

| **Item** | **Authors’ judgment** | **Risk of bias** | **Applicability concerns** |
| --- | --- | --- | --- |
| **Domain 1: Patient selection**  Risk of bias: Could the selection of patients have introduced bias?  Applicability: Are there concerns that the included patients and setting do not match the review question? | | | |
| Was a consecutive or random sample of patients enrolled? | Unclear |  |  |
| Was a case-control design avoided? | Yes |  |  |
| Did the study avoid inappropriate exclusions? | Unclear (no clear inclusion/exclusion criteria provided) |  |  |
| Judgment on risk of bias and applicability concerns |  | Unclear | Low |
| **Domain 2: Index test**  Risk of Bias: Could the conduct or interpretation of the index test have introduced bias?  Applicability: Are there concerns that the index test, its conduct, or interpretation differ from the review question? | | | |
| Were the index test results interpreted without knowledge of the results of the reference standard? | Unclear |  |  |
| If a threshold was used, was it pre-specified?  (always yes for NxTAG) | Yes |  |  |
| Judgment on risk of bias and applicability concerns |  | Low (unblinding unlikely to introduce interpretation bias) | Low |
| **Domain 3: Comparator test**  Risk of Bias: Could the comparator test, its conduct, or its interpretation have introduced bias?  Applicability: Are there concerns that the target condition as defined by the comparator test does not match the question? | | | |
| Is the comparator test likely to correctly classify the target condition? | Yes |  |  |
| Were the comparator test results interpreted without knowledge of the results of the index tests? | Unclear |  |  |
| Judgment on risk of bias and applicability concerns |  | Low (unblinding unlikely to introduce interpretation bias) | Low |
| **Domain 4: Flow and timing**  Risk of Bias: Could the patient flow have introduced bias? | | | |
| Was there an appropriate interval between index test and comparator test? | Unclear |  |  |
| Did all patients receive a comparator test? | Yes |  |  |
| Did all patients receive the same comparator test? | Yes |  |  |
| Were all patients included in the analysis? | Yes |  |  |
| Judgment on risk of bias and applicability concerns |  | Unclear | Not applicable |

**Table S5A. Esposito 2016: study characteristics**

| **Participant sampling** | Cross-sectional survey: analysis of samples collected during one month and retrospective analysis of stored samples | |
| --- | --- | --- |
| **Participant characteristics and setting** | **Country** | Italy |
|  | **Study centres** | Pediatric highly intensive care unit (PICU) of the Fondazione IRCCS Ca’ Granda Ospedale Maggiore Policlinico, Milan |
|  | **Study dates** | February 2015 |
|  | **Inclusion/exclusion criteria** | (A) Children admitted to PICU with respiratory tract infections, otherwise healthy  (B) Samples stored from children with community-acquired pneumonia that were positive for *M. pneumoniae* |
|  | **Sample size** | 185 samples (142 (A) + 43 (B)) |
|  | **Age** | Children; median age not reported |
|  | **Sex** | Not reported |
|  | **Respiratory tract infection symptoms** | Part of the inclusion criteria |
|  | **Febrile patients** | Not reported |
|  | **Inpatient/outpatient** | (A) Inpatients  (B) Not reported |
|  | **Samples collected** | Nasopharyngeal swabs |
| **Index test** | NxTAG-RPP | |
| **Comparator tests** | **1^st^ comparator test** | Luminex Respiratory Virus Panel (RVP) Fast Assay v2, produced by Luminex Molecular Diagnostics, Inc., Toronto, ON, Canada |
|  | **2^nd^ comparator test** | Singleplex RT-PCR for RSV A and B, hRV, hMPV, hADV and *M. pneumoniae.* Note that PCR was not performed for the detection of influenza virus. |
| **Flow and timing** | **Duration of sample storage** | (A) Not reported, but probably <6 months based on time between sample collection and submission of their manuscript  (B) Not reported |
|  | **Intervention and comparator tests performed in same sample** | Yes |
|  | **Missing data** | RT-PCR analyses were not available for 42 children  Luminex RVP Fast Assay v2 analyses produced “undetermined” results for 1 case  Although Luminex RVP Fast Assay v2 detects influenza, findings on influenza compared to NxTAG RPP were not reported |
| **Notes** | **Source of funding** | Grant from the Italian Ministry of Health |
|  | **Declared conflict of interests** | Authors declared no conflict of interest and specified that “Luminex Molecular Diagnostics Inc. did not participate in this study” |

**Table S5B. Esposito 2016: methodological quality (QUADAS-2)**

| **Item** | **Authors’ judgement** | **Risk of bias** | **Applicability concerns** |
| --- | --- | --- | --- |
| **Domain 1: Patient selection**  Risk of bias: Could the selection of patients have introduced bias?  Applicability: Are there concerns that the included patients and setting do not match the review question? | | | |
| Was a consecutive or random sample of patients enrolled? | Yes for 142 samples, unclear for 43 samples |  |  |
| Was a case-control design avoided? | Yes |  |  |
| Did the study avoid inappropriate exclusions? | Unclear |  |  |
| Judgment on risk of bias and applicability concerns |  | Unclear | Low |
| **Domain 2: Index test**  Risk of Bias: Could the conduct or interpretation of the index test have introduced bias?  Applicability: Are there concerns that the index test, its conduct, or interpretation differ from the review question? | | | |
| Were the index test results interpreted without knowledge of the results of the reference standard? | Unclear |  |  |
| If a threshold was used, was it pre-specified?  (always yes for NxTAG) | Yes |  |  |
| Judgment on risk of bias and applicability concerns |  | Low (unblinding unlikely to introduce interpretation bias) | Low |
| **Domain 3: Comparator test**  Risk of Bias: Could the comparator test, its conduct, or its interpretation have introduced bias?  Applicability: Are there concerns that the target condition as defined by the comparator test does not match the question? | | | |
| Is the comparator test likely to correctly classify the target condition? | Yes |  |  |
| Were the comparator test results interpreted without knowledge of the results of the index tests? | Unclear |  |  |
| Judgment on risk of bias and applicability concerns |  | Low (unblinding unlikely to introduce interpretation bias) | Low |
| **Domain 4: Flow and timing**  Risk of Bias: Could the patient flow have introduced bias? | | | |
| Was there an appropriate interval between index test and comparator test? | Unclear |  |  |
| Did all patients receive a comparator test? | Yes |  |  |
| Did all patients receive the same comparator test? | Yes for reference test 1  No for reference test 2 |  |  |
| Were all patients included in the analysis? | Yes for RSV  No for influenza |  |  |
| Judgment on risk of bias and applicability concerns |  | Unclear | Not applicable |

**Table S6A. Gonsalves 2019: study characteristics**

| **Participant sampling** | Cross sectional study with prospective data collection | |
| --- | --- | --- |
| **Participant characteristics and setting** | **Country** | USA and Canada |
|  | **Study centres** | 4 clinical sites |
|  | **Study dates** | January to April 2014 (934 samples) and January to March 2015 (1198 samples) |
|  | **Inclusion/exclusion criteria** | Nasopharyngeal swab specimens from children and adults hospitalized, admitted to a hospital emergency department, visited an outpatient clinic, or resided at a long-term care facility with clinical signs and symptoms of respiratory tract infection |
|  | **Sample size** | 2132 samples |
|  | **Age** | Children and adults  0-1 year: 453 (21.2%); 1-5 years: 250 (11.7%); 5-21 years: 353 (16.6%); 21-65 years: 584 (27.4%); > 65 years: 492 (23.1%) |
|  | **Sex** | 1110 (52.1%) females |
|  | **Respiratory tract infection symptoms** | Part of the inclusion criteria |
|  | **Febrile patients** | Not reported |
|  | **Inpatient/outpatient** | Outpatients: 554 (26.0%); hospitalized: 1060 (49.7%); emergency department: 518 (24.3%) |
|  | **Samples collected** | Nasopharyngeal swabs |
| **Index test** | NxTAG-RPP | |
| **Comparator tests** | **1^st^ comparator test** | xTAG RVP **OR** bidirectional sequencing |
|  | **2^nd^ comparator test** | For discordant results only: FDA-cleared RT-PCR assay routinely used at the clinical sites (BioFire FilmArray RP or xTAG RVP) or by bidirectional sequencing using analytically validated primers that targeted genomic regions distinct from those targeted by NxTAG RPP |
| **Flow and timing** | **Duration of sample storage** | < 1 month.  Authors stated that “Specimens can be stored at 2–8 °C for up to 7 days after collection in Universal Transport Media or equivalent. If the specimen is not going to be tested within 7 days of collection, it may be stored at ≤−70 °C for up to 12 months.” |
|  | **Intervention and comparator tests performed in same sample** | Yes |
|  | **Missing data** | 101 invalid results for one or more analytes during NxTAG testing for all viruses tested; 9 for Influenza AH3, 8 for Influenza B, 7 for RSV-A, 9 for RSV-B.  Invalid results were due to external control failure or non-specific signals in external controls (3.8%), low bead count (0.5%), internal control failure (0.3%), and inconclusive results due to abnormal signals (0.1%). The frequency of invalid results was evenly distributed among the clinical sites.  Available residual specimens were re-run with NxTAG RPP. |
| **Notes** | **Source of funding** | Not reported |
|  | **Declared conflict of interests** | Not reported, but two authors are affiliated with Luminex Corporation |

**Table S6B. Gonsalves 2019: methodological quality (QUADAS-2)**

| **Item** | **Authors’ judgment** | **Risk of bias** | **Applicability concerns** |
| --- | --- | --- | --- |
| **Domain 1: Patient selection**  Risk of bias: Could the selection of patients have introduced bias?  Applicability: Are there concerns that the included patients and setting do not match the review question? | | | |
| Was a consecutive or random sample of patients enrolled? | Unclear |  |  |
| Was a case-control design avoided? | Yes |  |  |
| Did the study avoid inappropriate exclusions? | Unclear |  |  |
| Judgment on risk of bias and applicability concerns |  | Unclear | Low |
| **Domain 2: Index test**  Risk of Bias: Could the conduct or interpretation of the index test have introduced bias?  Applicability: Are there concerns that the index test, its conduct, or interpretation differ from the review question? | | | |
| Were the index test results interpreted without knowledge of the results of the reference standard? | Unclear |  |  |
| If a threshold was used, was it pre-specified?  (always yes for NxTAG) | Yes |  |  |
| Judgment on risk of bias and applicability concerns |  | Low (unblinding unlikely to introduce interpretation bias) | Low |
| **Domain 3: Comparator test**  Risk of Bias: Could the comparator test, its conduct, or its interpretation have introduced bias?  Applicability: Are there concerns that the target condition as defined by the comparator test does not match the question? | | | |
| Is the comparator test likely to correctly classify the target condition? | Yes |  |  |
| Were the comparator test results interpreted without knowledge of the results of the index tests? | Unclear |  |  |
| Judgment on risk of bias and applicability concerns |  | Low (unblinding unlikely to introduce interpretation bias) | Low |
| **Domain 4: Flow and timing**  Risk of Bias: Could the patient flow have introduced bias? | | | |
| Was there an appropriate interval between index test and comparator test? | Yes |  |  |
| Did all patients receive a comparator test? | Yes |  |  |
| Did all patients receive the same comparator test? | No |  |  |
| Were all patients included in the analysis? | Yes |  |  |
| Judgment on risk of bias and applicability concerns |  | High | Not applicable |

**Table S7A. Lee 2017: study characteristics**

| **Participant sampling** | Retrospective data collection | |
| --- | --- | --- |
| **Participant characteristics and setting** | **Country** | Singapore |
|  | **Study centres** | Molecular Diagnosis Centre of the Singapore National University Hospital |
|  | **Study dates** | May to December 2015 |
|  | **Inclusion/exclusion criteria** | Not reported |
|  | **Sample size** | 142 samples |
|  | **Age** | Not reported |
|  | **Sex** | Not reported |
|  | **Respiratory tract infection symptoms** | Not reported |
|  | **Febrile patients** | Not reported |
|  | **Inpatient/outpatient** | Not reported |
|  | **Samples collected** | “Clinical respiratory samples” |
| **Index test** | NxTAG-RPP | |
| **Comparator tests** | **1^st^ comparator test** | xTAG RVP FAST v2, Luminex, manufactured by Luminex Corp. (Austin, TX, USA) |
|  | **2^nd^ comparator test** | For discordant results only: in-house respiratory panel |
| **Flow and timing** | **Duration of sample storage** | Not reported, but probably <12 months based on time between sample collection and submission of their manuscript |
|  | **Intervention and comparator tests performed in same sample** | Yes |
|  | **Missing data** | None |
| **Notes** | **Source of funding** | Not reported |
|  | **Declared conflict of interests** | Authors declared no conflict of interest |

**Table S7B. Lee 2017: methodological quality (QUADAS-2)**

| **Item** | **Authors’ judgment** | **Risk of bias** | **Applicability concerns** |
| --- | --- | --- | --- |
| **Domain 1: Patient selection**  Risk of bias: Could the selection of patients have introduced bias?  Applicability: Are there concerns that the included patients and setting do not match the review question? | | | |
| Was a consecutive or random sample of patients enrolled? | No |  |  |
| Was a case-control design avoided? | Yes |  |  |
| Did the study avoid inappropriate exclusions? | Unclear |  |  |
| Judgment on risk of bias and applicability concerns |  | High | Unclear (unclear characteristics of participants) |
| **Domain 2: Index test**  Risk of Bias: Could the conduct or interpretation of the index test have introduced bias?  Applicability: Are there concerns that the index test, its conduct, or interpretation differ from the review question? | | | |
| Were the index test results interpreted without knowledge of the results of the reference standard? | Unclear |  |  |
| If a threshold was used, was it pre-specified?  (always yes for NxTAG) | Yes |  |  |
| Judgment on risk of bias and applicability concerns |  | Low (unblinding unlikely to introduce interpretation bias) | Low |
| **Domain 3: Comparator test**  Risk of Bias: Could the comparator test, its conduct, or its interpretation have introduced bias?  Applicability: Are there concerns that the target condition as defined by the comparator test does not match the question? | | | |
| Is the comparator test likely to correctly classify the target condition? | Yes |  |  |
| Were the comparator test results interpreted without knowledge of the results of the index tests? | Yes |  |  |
| Judgment on risk of bias and applicability concerns |  | Low | Low |
| **Domain 4: Flow and timing**  Risk of Bias: Could the patient flow have introduced bias? | | | |
| Was there an appropriate interval between index test and comparator test? | Unclear |  |  |
| Did all patients receive a comparator test? | Yes |  |  |
| Did all patients receive the same comparator test? | Yes |  |  |
| Were all patients included in the analysis? | Yes |  |  |
| Judgment on risk of bias and applicability concerns |  | Unclear | Not applicable |

**Table S8A. Locher 2019: study characteristics**

| **Participant sampling** | Cross sectional study with (A) retrospective data analysis on archived samples, and (B) samples collected prospectively | |
| --- | --- | --- |
| **Participant characteristics and setting** | **Country** | Canada |
|  | **Study centres** | Diagnostic microbiology laboratory serving an acute tertiary care center in Vancouver |
|  | **Study dates** | (A) Archived samples collected between December 2015 and November 2018; FA RP testing between September 2016 and February 2019  (B) Prospective samples collected between December 2016 and May 2017 |
|  | **Inclusion/exclusion criteria** | (A) Not reported  (B) Patients with suspected acute respiratory tract infection from the bone marrow transplant unit, respiratory/thoracic unit, intensive care unit, and other specific locations chosen because they typically house immunocompromised patients and patients with underlying chronic lung disease |
|  | **Sample size** | 133 samples |
|  | **Age** | Adults  (A) Mean age 53 years (SD 14.8)  (B) Mean age 56 years (SD 14.6) |
|  | **Sex** | Not reported |
|  | **Respiratory tract infection symptoms** | (A) Not reported  (B) Part of the inclusion criteria |
|  | **Febrile patients** | Not reported |
|  | **Inpatient/outpatient** | Both inpatients and outpatients.  (A) outpatient bronchoscopy suite (n=33), intensive care unit (n=12), respiratory/thoracic unit (n=10), respiratory ambulatory unit (n=7), bone marrow transplant unit (n=4), medicine unit (n=4), tuberculosis unit (n=1), pre-admitting center (n=1) and unknown (n=15).  (B) intensive care unit (n=22), respiratory/thoracic unit (n=13), bone marrow transplant unit (n=11). |
|  | **Samples collected** | Bronchoscopy specimens: 120 bronchoalveolar lavages, 9 bronchial aspirates, 4 bronchial washes.  All testing was performed on neat undiluted bronchoscopy specimens without pre-treatment of mucoid specimens. |
| **Index test** | NxTAG-RPP (Luminex Molecular Diagnostics, Toronto, Canada) | |
| **Comparator tests** | **1^st^ comparator test** | xTAG RVP FAST v2, Luminex, manufactured by Luminex Corp. (Austin, TX, USA) |
|  | **2^nd^ comparator test** | Specimens with discordant viral targets were re-tested by NxTAG RPP to determine if degradation of the analyte had occurred during storage of these archived samples that would account for the discordant results.  Consensus was defined as a minimum of two out of three results being in agreement (FA RP, initial NxTAG RPP and repeat NxTAG RPP/LD PCR). |
| **Flow and timing** | **Duration of sample storage** | (A) Samples stored at -70 °C for long term storage  (B) Samples were kept at 4 °C for storage less than one week |
|  | **Intervention and comparator tests performed in same sample** | Yes |
|  | **Missing data** | 2 samples  For 1 case: NxTAG RPP detected BoV and human hRV/EV while the FA RP result was negative. Since BoV is not a FA RP target, this result was disregarded and only the hRV/EV result was considered discordant.  For 1 case: specimen discordant for PIV 4 (FA RP negative, NxTAG RPP positive) could not be repeated due to insufficient sample and was excluded from analysis |
| **Notes** | **Source of funding** | See below |
|  | **Declared conflict of interests** | Biomerieux Canada supplied the BioFire equipment and FA RP kits, but otherwise had no role in study design, implementation and interpretation of results or manuscript preparation. |

**Table S8B. Locher 2019: methodological quality (QUADAS-2)**

| **Item** | **Authors’ judgement** | **Risk of bias** | **Applicability concerns** |
| --- | --- | --- | --- |
| **Domain 1: Patient selection**  Risk of bias: Could the selection of patients have introduced bias?  Applicability: Are there concerns that the included patients and setting do not match the review question? | | | |
| Was a consecutive or random sample of patients enrolled? | No (“randomly collected” for (B) with no details on the method of randomization, and no for (A)) |  |  |
| Was a case-control design avoided? | Yes |  |  |
| Did the study avoid inappropriate exclusions? | Unclear |  |  |
| Judgment on risk of bias and applicability concerns |  | High | Low |
| **Domain 2: Index test**  Risk of Bias: Could the conduct or interpretation of the index test have introduced bias?  Applicability: Are there concerns that the index test, its conduct, or interpretation differ from the review question? | | | |
| Were the index test results interpreted without knowledge of the results of the reference standard? | Yes (for A: 83)  Unclear for B |  |  |
| If a threshold was used, was it pre-specified?  (always yes for NxTAG) | Yes |  |  |
| Judgment on risk of bias and applicability concerns |  | Low | Low |
| **Domain 3: Comparator test**  Risk of Bias: Could the comparator test, its conduct, or its interpretation have introduced bias?  Applicability: Are there concerns that the target condition as defined by the comparator test does not match the question? | | | |
| Is the comparator test likely to correctly classify the target condition? | Yes |  |  |
| Were the comparator test results interpreted without knowledge of the results of the index tests? | No (for A: 83)  Unclear for B |  |  |
| Judgment on risk of bias and applicability concerns |  | Low | Low |
| **Domain 4: Flow and timing**  Risk of Bias: Could the patient flow have introduced bias? | | | |
| Was there an appropriate interval between index test and comparator test? | Unclear |  |  |
| Did all patients receive a comparator test? | Yes |  |  |
| Did all patients receive the same comparator test? | Yes |  |  |
| Were all patients included in the analysis? | Yes (except 2 with reason given) |  |  |
| Judgment on risk of bias and applicability concerns |  | Unclear | Not applicable |

**Table S9A. Sails 2017: study characteristics**

| **Participant sampling** | Cross sectional study with retrospective data analysis on stored samples, selected based on the results of the microbiological test “to ensure that the number of virus-positive samples would be higher than in randomly selected prospectively collected samples” | |
| --- | --- | --- |
| **Participant characteristics and setting** | **Country** | United Kingdom |
|  | **Study centres** | Not reported, but affiliation of authors from Public Health England Newcastle Laboratory and Newcastle upon Tyne Hospitals NHS Foundation Trust, Freeman Hospital. |
|  | **Study dates** | October 204 to February 2016 |
|  | **Inclusion/exclusion criteria** | “Symptomatic patients either on presentation at their GP surgery or in hospital” |
|  | **Sample size** | 314 samples |
|  | **Age** | Not reported |
|  | **Sex** | Not reported |
|  | **Respiratory tract infection symptoms** | “Symptomatic patients” with no further details reported |
|  | **Febrile patients** | Not reported |
|  | **Inpatient/outpatient** | Outpatients, and inpatient unclear (“in hospital”, but does not say if admitted patients) |
|  | **Samples collected** | Nasopharyngeal secretions (n=122), throat swabs (n=53), endotracheal secretions (n=47), combined nose and throat swabs (n=41), sputum (n=24), bronchoalveolar lavages (n=17), nasal swabs (n=8), bronchial secretions (n=1) and eye swabs (n=1) |
| **Index test** | NxTAG-RPP | |
| **Comparator tests** | **1^st^ comparator test** | In-house multiplex RT-PCR panel |
|  | **2^nd^ comparator test** | For discordant results: PCR (direct sequencing of specific pathogen gene targets with PCR and Sanger sequencing primers designed not to overlap with the primers used in the NxTAG RPP assay) |
| **Flow and timing** | **Duration of sample storage** | Stored at -80ºC for up to 12 months |
|  | **Intervention and comparator tests performed in same sample** | Yes |
|  | **Missing data** | 2 samples, due to invalid results in NxTAG-RPP |
| **Notes** | **Source of funding** | Luminex Molecular Diagnostics provided the NxTAG-RPP reagents and MagPIX instrument |
|  | **Declared conflict of interests** | Authors declared no conflict of interest |

**Table S9B. Sails 2017: methodological quality (QUADAS-2)**

| **Item** | **Authors’ judgment** | **Risk of bias** | **Applicability concerns** |
| --- | --- | --- | --- |
| **Domain 1: Patient selection**  Risk of bias: Could the selection of patients have introduced bias?  Applicability: Are there concerns that the included patients and setting do not match the review question? | | | |
| Was a consecutive or random sample of patients enrolled? | No (samples selected based on positive results of reference test) |  |  |
| Was a case-control design avoided? | Yes |  |  |
| Did the study avoid inappropriate exclusions? | Unclear |  |  |
| Judgment on risk of bias and applicability concerns |  | High | Unclear (unclear characteristics of participants) |
| **Domain 2: Index test**  Risk of Bias: Could the conduct or interpretation of the index test have introduced bias?  Applicability: Are there concerns that the index test, its conduct, or interpretation differ from the review question? | | | |
| Were the index test results interpreted without knowledge of the results of the reference standard? | Unclear (reference test was performed first, but unclear whether the interpreter knew the test result) |  |  |
| If a threshold was used, was it pre-specified?  (always yes for NxTAG) | Yes |  |  |
| Judgment on risk of bias and applicability concerns |  | Low (unblinding unlikely to introduce interpretation bias) | Low |
| **Domain 3: Comparator test**  Risk of Bias: Could the comparator test, its conduct, or its interpretation have introduced bias?  Applicability: Are there concerns that the target condition as defined by the comparator test does not match the question? | | | |
| Is the comparator test likely to correctly classify the target condition? | Yes |  |  |
| Were the comparator test results interpreted without knowledge of the results of the index tests? | Yes |  |  |
| Judgment on risk of bias and applicability concerns |  | Low | Low |
| **Domain 4: Flow and timing**  Risk of Bias: Could the patient flow have introduced bias? | | | |
| Was there an appropriate interval between index test and comparator test? | Unclear (up to 12 months) |  |  |
| Did all patients receive a comparator test? | Yes |  |  |
| Did all patients receive the same comparator test? | Yes |  |  |
| Were all patients included in the analysis? | Yes |  |  |
| Judgment on risk of bias and applicability concerns |  | Unclear | Not applicable |

**Table S10A. Tang 2016: study characteristics**

| **Participant sampling** | Cross sectional study with retrospective data analysis on remnant samples, some collected consecutively, others selected based on positive viral testing on reference test | |
| --- | --- | --- |
| **Participant characteristics and setting** | **Country** | USA |
|  | **Study centres** | Memorial Sloan-Kettering Cancer Center (MSKCC) |
|  | **Study dates** | 25 September to 1 October 2013, and 2013-2014 and 2014-2015 respiratory virus seasons |
|  | **Inclusion/exclusion criteria** | Patients with symptoms of respiratory tract infection according to the study objective, but no clear inclusion criteria reported |
|  | **Sample size** | 404 samples |
|  | **Age** | Not reported |
|  | **Sex** | Not reported |
|  | **Respiratory tract infection symptoms** | Part of the study objective, not clearly reported |
|  | **Febrile patients** | Not reported |
|  | **Inpatient/outpatient** | Not reported |
|  | **Samples collected** | Nasopharyngeal swabs |
| **Index test** | NxTAG-RPP | |
| **Comparator tests** | **1^st^ comparator test** | FilmArray Respiratory Panel |
|  | **2^nd^ comparator test** | For discordant results: RT-PCR (bidirectional sequencing) |
| **Flow and timing** | **Duration of sample storage** | Stored at -80ºC for unclear duration (up to 3 years?) |
|  | **Intervention and comparator tests performed in same sample** | Yes |
|  | **Missing data** | 2 samples, due to insufficient sample to repeat test for discordant results |
| **Notes** | **Source of funding** | Research agreement between the MSKCC and the Luminex Corporation and by an NIH/NCI Cancer Center Support Grant P30 |
|  | **Declared conflict of interests** | 3 authors are employees of Luminex Corporation, the commercial manufacturer of the NxTAG RPP |

**Table S10B. Tang 2016: methodological quality (QUADAS-2)**

| **Item** | **Authors’ judgment** | **Risk of bias** | **Applicability concerns** |
| --- | --- | --- | --- |
| **Domain 1: Patient selection**  Risk of bias: Could the selection of patients have introduced bias?  Applicability: Are there concerns that the included patients and setting do not match the review question? | | | |
| Was a consecutive or random sample of patients enrolled? | 194 consecutive samples but unclear on patient selection  No for 206 (known positive) |  |  |
| Was a case-control design avoided? | Yes |  |  |
| Did the study avoid inappropriate exclusions? | Unclear |  |  |
| Judgment on risk of bias and applicability concerns |  | High | Low |
| **Domain 2: Index test**  Risk of Bias: Could the conduct or interpretation of the index test have introduced bias?  Applicability: Are there concerns that the index test, its conduct, or interpretation differ from the review question? | | | |
| Were the index test results interpreted without knowledge of the results of the reference standard? | No |  |  |
| If a threshold was used, was it pre-specified?  (always yes for NxTAG) | Yes |  |  |
| Judgment on risk of bias and applicability concerns |  | Low (unblinding unlikely to introduce interpretation bias) | Low |
| **Domain 3: Comparator test**  Risk of Bias: Could the comparator test, its conduct, or its interpretation have introduced bias?  Applicability: Are there concerns that the target condition as defined by the comparator test does not match the question? | | | |
| Is the comparator test likely to correctly classify the target condition? | Yes |  |  |
| Were the comparator test results interpreted without knowledge of the results of the index tests? | Yes for 206  Unclear for 194 |  |  |
| Judgment on risk of bias and applicability concerns |  | Low (unblinding unlikely to introduce interpretation bias) | Low |
| **Domain 4: Flow and timing**  Risk of Bias: Could the patient flow have introduced bias? | | | |
| Was there an appropriate interval between index test and comparator test? | Unclear |  |  |
| Did all patients receive a comparator test? | Yes |  |  |
| Did all patients receive the same comparator test? | Yes |  |  |
| Were all patients included in the analysis? | Yes (2 samples excluded with reason) |  |  |
| Judgment on risk of bias and applicability concerns |  | Unclear | Not applicable |

**Table S11. Findings of Luminex NxTAG RPP™ against comparator test for detecting RSV (RSV-A or RSV-B)**

| **Study ID** | **Copmarator test** | **I+/C+** | **I+/C-** | **I-/C+** | **I-/C-** | **Notes** |
| --- | --- | --- | --- | --- | --- | --- |
| Beckmann 2016 | RespiFinder-221 | 26 | 0 | 0 | 256 | Authors only provided disaggregated findings for RSV-A and RSV-B. They reported no co-infection RSV-A/RSV-B; therefore we could pool the data for RSV A+B |
| Brotons 2016 | Anyplex II RV16 | NA | NA | NA | NA | By contacting the authors, they informed that data was collected by virus subtype and that there is no information available on co-infection that would allow to pool the data for RSV-A and RSV-B |
| Chan 2017 | RT-PCR + DFA | NA | NA | NA | NA | We contacted the authors to request these data with no reply at the time of publication |
| Chen 2016 | BioFire FilmArray RP | 40 | 6 | 0 | 238 | - |
| Esposito 2016 | xTAG RVP FAST v2 | 69 | 11 | 0 | 104 | “Undetermined” result for 1 child |
|  | RT-PCR | 55 | 11 | 0 | 77 | RT-PCR analyses were not available for 42 children |
| Gonsalves 2019 | xTAG RVP or bidirectional sequencing | 204 | 27 | 2 | 4015 | - |
| Lee 2017 | xTAG RVP FAST v2 | 9 | 0 | 0 | 133 | PCR for discordant results was planned but there were no discordant results between the index and comparator tests in the detection of RSV, so in-house PCR was not required. |
| Locher 2019 | BioFire FilmArray RP | 6 | 0 | 0 | 125 | There were no discordant results on the detection of RSV |
| Sails 2017 | In-house multiplex RT-PCR panel | 47 | 1 | 0 | 264 | Authors were contacted. They provided these aggregated data, which were not included in the published manuscript.  PCR was done for discordant results, leading to 48 I+/C+ and 0 I+/C- |
| Tang 2016 | FilmArray RP | 14 | 1 | 0 | 387 | - |

Abbreviations: C: comparator; I: index test; NA: not available.

**Table S12. Findings of Luminex NxTAG RPP™ against comparator test for detecting RSV-A and RSV-B**

| **Study ID** | **Comparator test** | **RSV-A** | | | | **RSV-B** | | | |
| --- | --- | --- | --- | --- | --- | --- | --- | --- | --- |
|  |  | **I+/C+** | **I+/C-** | **I-/C+** | **I-/C-** | **I+/C+** | **I+/C-** | **I-/C+** | **I-/C-** |
| Beckmann 2016 | RespiFinder-221 | 13 | 0 | 0 | 269 | 13 | 0 | 0 | 269 |
| Brotons 2016 | Anyplex II RV16 | 19 | 0 | 0 | 300 | 24 | 4 | 1 | 290 |
| Chan 2017 | RT-PCR + DFA | 9 | 1 | 0 | 123 | 2 | 0 | 0 | 131 |
| Chen 2016 | BioFire FilmArray RP | NA | NA | NA | NA | NA | NA | NA | NA |
| Esposito 2016 | xTAG RVP FAST v2 | NA | NA | NA | NA | NA | NA | NA | NA |
|  | RT-PCR | 33 | 3 | 0 | 107 | 23 | 8 | 0 | 112 |
| Gonsalves 2019 | xTAG RVP or bidirectional sequencing | 73 | 15 | 0 | 2037 | 131 | 12 | 2 | 1978 |
| Lee 2017 | xTAG RVP FAST v2 | NA | NA | NA | NA | NA | NA | NA | NA |
| Locher 2019 | BioFire FilmArray RP | NA | NA | NA | NA | NA | NA | NA | NA |
| Sails 2017 | In-house multiplex RT-PCR panel | 44 | 1 | 0 | 267 | 3 | 0 | 0 | 309 |
| Tang 2016 | FilmArray RP | NA | NA | NA | NA | NA | NA | NA | NA |

Abbreviations: C: comparator; I: index test; NA: not available.

**Table S13. Findings of Luminex NxTAG RPP™ for diagnosing influenza A/B viruses**

| **Study ID** | **Comparator test** | **Influenza A virus** | | | | | **Influenza B virus** | | | |
| --- | --- | --- | --- | --- | --- | --- | --- | --- | --- | --- |
|  |  | **Subtypes** | **I+/C+** | **I+/C-** | **I-/C+** | **I-/C-** | **I+/C+** | **I+/C-** | **I-/C+** | **I-/C-** |
| Beckmann 2016 | RespiFinder-221 | A^a^  AH1  AH3 | 11  1  10 | 0  0  0 | 0  0  0 | 271  281  272 | 19 | 0 | 0 | 263 |
| Brotons 2016 | Anyplex II RV16 | A | 9 | 1 | 1 | 308 | 1 | 0 | 0 | 318 |
| Chan 2017 | RT-PCR + DFA | A  AH1  AH1pdm09  AH3 | 16  0  3  12 | 1  0  1  0 | 0  0  0  0 | 116  133  129  121 | 10 | 0 | 0 | 123 |
| Chen 2016 | BioFire FilmArray RP | A^b^  AH1pdm09  AH3 | 49  14  34 | 0  0  1 | 0  1  0 | 235  269  249 | 20 | 0 | 0 | 264 |
| Esposito 2016 | xTAG RVP FAST v2 | NA | NA | NA | NA | NA | NA | NA | NA | NA |
| Gonsalves 2019 | xTAG RVP or bidirectional sequencing | A  AH1  AH3 | 259  21  203 | 37  20  45 | 14  0  3 | 1822  2091  1872 | 87 | 14 | 4 | 2019 |
| Lee 2017 | xTAG RVP FAST v2 | A  AH1  AH3 | 12  1  3 | 0  0  8 | 0  0  0 | 130  141  131 | 2 | 0 | 0 | 140 |
| Locher 2019 | BioFire FilmArray RP | A | 5 | 1 | 1 | 124 | 4 | 0 | 1 | 126 |
| Sails 2017 | In-house multiplex RT-PCR panel | A^a^  AH1  AH3 | 39  2  37 | 0  0  0 | 1  0  1 | 272  310  274 | 4 | 0 | 0 | 308 |
| Tang 2016 | FilmArray RP | A^a^  AH1  AH3 | 42  19  23 | 1  1  0 | 1  1  0 | 358  381  379 | 12 | 1 | 0 | 389 |

Abbreviations: C: comparator; I: index test; NA: not available.

^a^The number of I+/C+, I+/C-, I-/C+ and I-/C- were only provided for influenza subtypes. We calculated the number of I+/C+, I+/C-, I-/C+ and I-/C- for influenza A virus from the influenza subtypes, assuming no co-infection with two influenza A subtypes.

^b^In addition, 3 samples from positive A/H7N9 (avian influenza) viral culture were tested with NxTAG. All of them were detected by the NxTAG-RPP influenza A matrix gene target. All the hemagglutinin subtyping gene targets (H1, H1pdm09, H3) included in the panel were negative for the 3 H7N9 samples.

# Additional file 1: figures


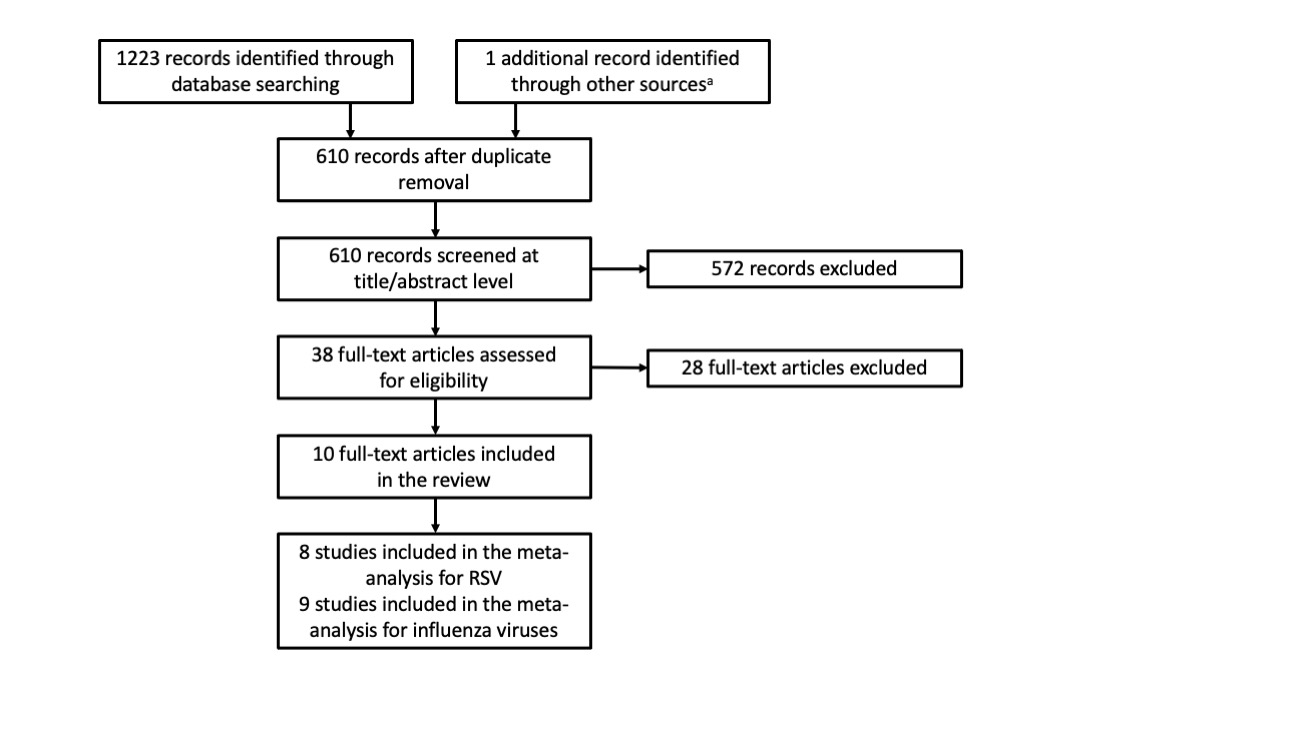


**Figure S1.** Flow diagram of literature review process for studies included in the systematic review and meta-analyses.

^a^This additional record was identified by the manufacturer and was included in the review (Gonsalves S, Mahony J, Rao A, Dunbar S, Juretschko S. Multiplexed detection and identification of respiratory pathogens using the NxTAG ® respiratory pathogen panel. Methods. 2019; 158:61–8.)


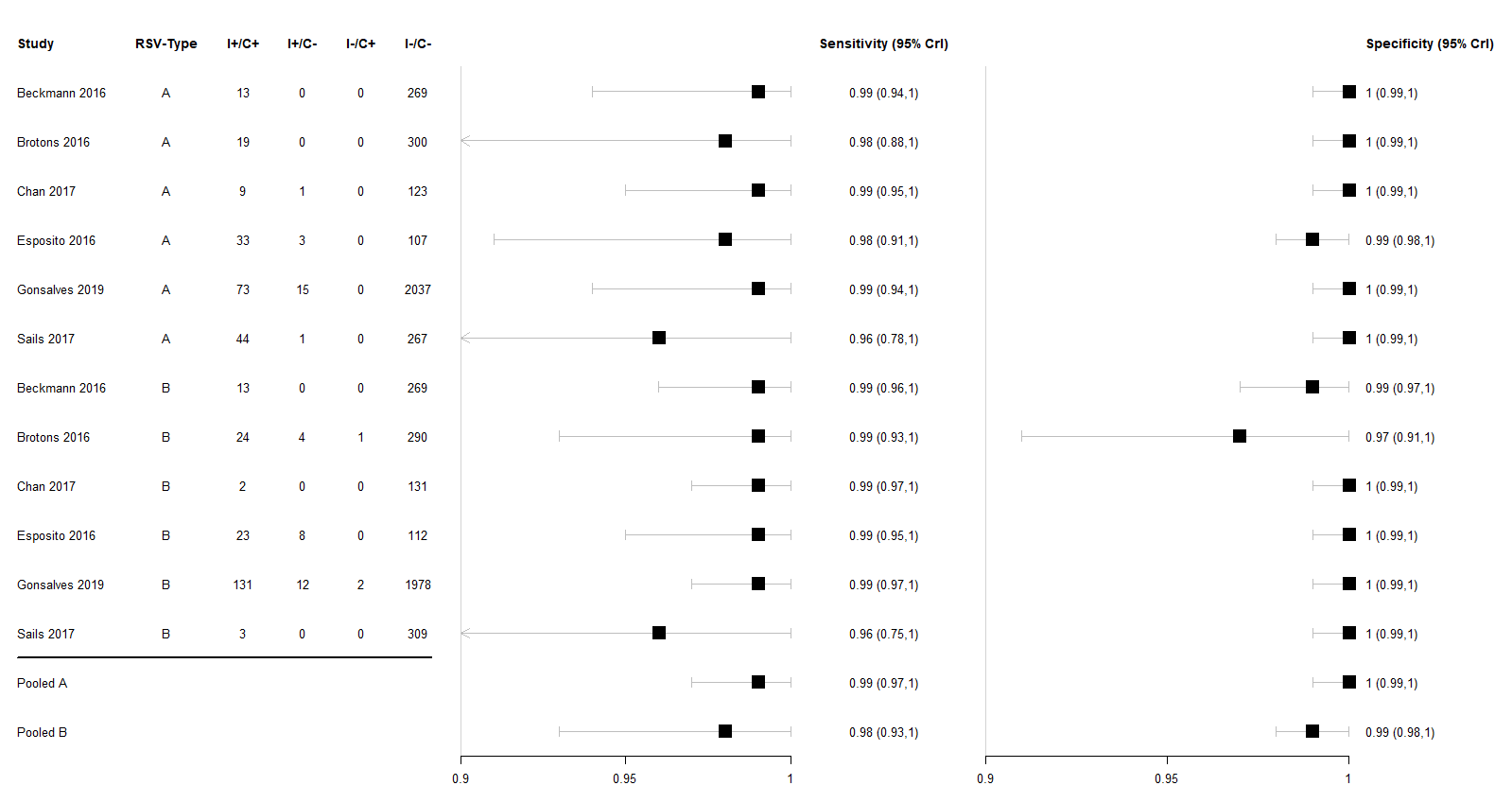


**Figure S2**. Forest plot of the sensitivity and specificity of the Luminex NxTAG RPP^TM^ versus comparators for detecting RSV-A and RSV-B with their 95% credible intervals.


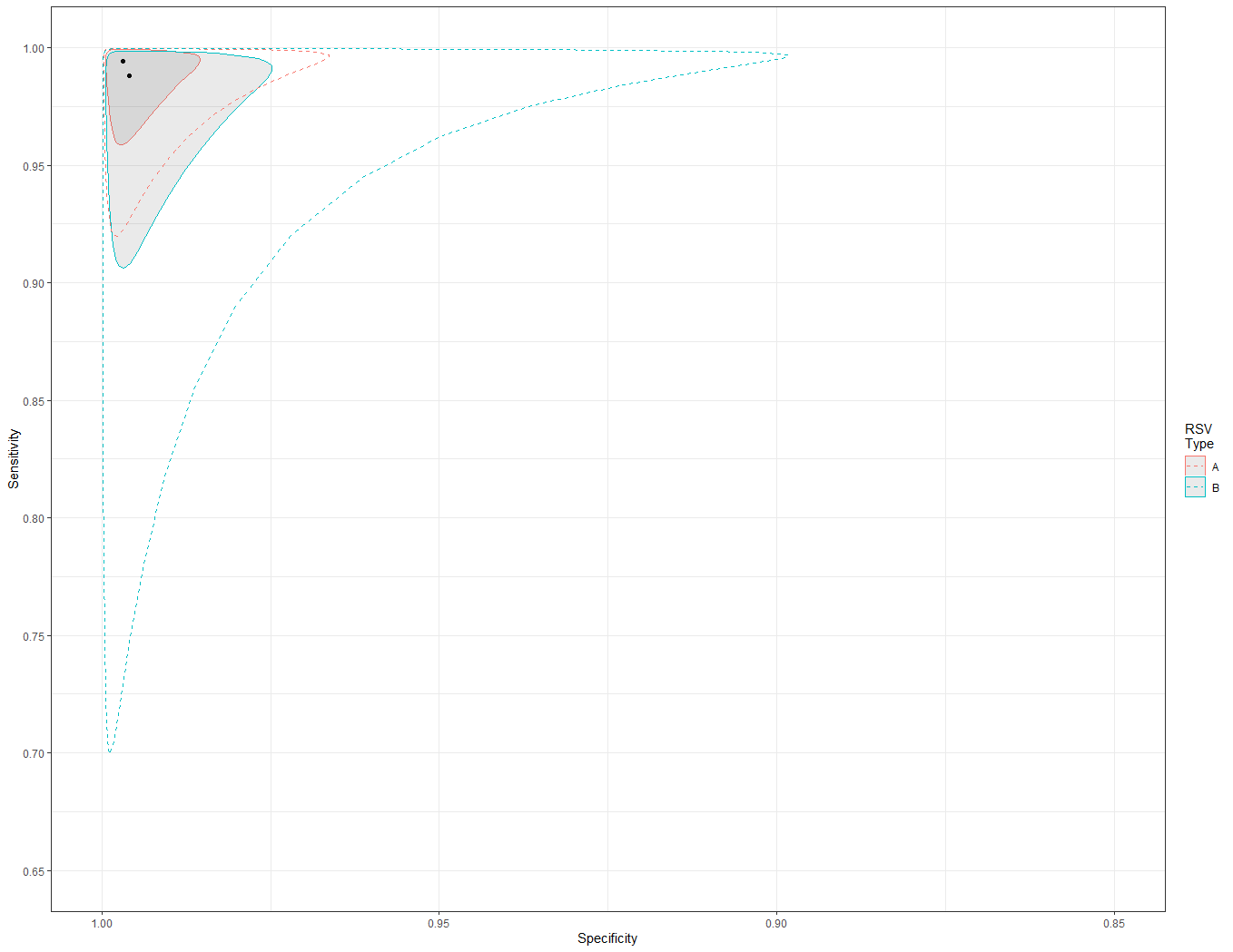


**Figure S3.** Pooled (shaded) and predicted (dashed) estimates and credible regions of Luminex NxTAG RPP^TM^ for detecting RSV-A and RSV-B. Predicted sensitivity for RSV-A and RSV-B was 0.99 (0.93–1) and 0.96 (0.66–1), respectively and specificity 0.99 (0.96–1) and 0.98 (0.87–1), respectively.
